# Dynamics of transcriptional (re)-programming of syncytial nuclei in developing muscles

**DOI:** 10.1186/s12915-017-0386-2

**Published:** 2017-06-09

**Authors:** Laetitia Bataillé, Hadi Boukhatmi, Jean-Louis Frendo, Alain Vincent

**Affiliations:** 1 Centre de Biologie du Développement (CBD), Centre de Biologie Intégrative (CBI), Université de Toulouse, CNRS, UPS, Toulouse, France; 2 Present address: Department of Physiology, Development and Neuroscience, University of Cambridge, Downing Street, Cambridge, CB2 3DY UK

**Keywords:** Myogenesis, Syncytia, Muscle identity, Realisation genes, Transcriptional regulation, *Drosophila*

## Abstract

**Background:**

A stereotyped array of body wall muscles enables precision and stereotypy of animal movements. In *Drosophila*, each syncytial muscle forms via fusion of one founder cell (FC) with multiple fusion competent myoblasts (FCMs). The specific morphology of each muscle, i.e. distinctive shape, orientation, size and skeletal attachment sites, reflects the specific combination of identity transcription factors (iTFs) expressed by its FC. Here, we addressed three questions: Are FCM nuclei naive? What is the selectivity and temporal sequence of transcriptional reprogramming of FCMs recruited into growing syncytium? Is transcription of generic myogenic and identity realisation genes coordinated during muscle differentiation?

**Results:**

The tracking of nuclei in developing muscles shows that FCM nuclei are competent to be transcriptionally reprogrammed to a given muscle identity, post fusion. In situ hybridisation to nascent transcripts for FCM, FC-generic and iTF genes shows that this reprogramming is progressive, beginning by repression of FCM-specific genes in fused nuclei, with some evidence that FC nuclei retain specific characteristics. Transcription of identity realisation genes is linked to iTF activation and regulated at levels of both transcription initiation rate and period of transcription. The generic muscle differentiation programme is activated independently.

**Conclusions:**

Transcription reprogramming of fused myoblast nuclei is progressive, such that nuclei within a syncytial fibre at a given time point during muscle development are heterogeneous with regards to specific gene transcription. This comprehensive view of the dynamics of transcriptional (re)programming of post-mitotic nuclei within syncytial cells provides a new framework for understanding the transcriptional control of the lineage diversity of multinucleated cells.

**Electronic supplementary material:**

The online version of this article (doi:10.1186/s12915-017-0386-2) contains supplementary material, which is available to authorized users.

## Background

The musculature of each animal species is composed of a complex array of body wall muscles that enable precise and stereotypic movements. Muscle formation involves fusion of mononucleated myoblasts. The coupling of muscle differentiation with multinucleation raises the general question of transcriptional (re)-programming of nuclei within a syncytium. Whereas the transcriptional control of generic aspects of myogenesis has been largely decrypted in vertebrates, elucidating the mechanisms that confer each muscle a specific morphology, i.e. distinctive shape and orientation, number of nuclei, size, skeleton attachment sites and innervation, remains a major challenge in myology research.

Because of its relative simplicity, the somatic musculature of the *Drosophila* larva is a classical model to approach generic and morphology aspects of muscle formation. Every abdominal hemisegment displays approximately 30 distinct body wall muscles, each corresponding to a single multinucleated fibre with a unique morphology [1]. Each muscle develops by fusion of one founder cell (FC) with a given number of fusion competent myoblasts (FCMs) [1]. FC-FCM fusion is driven by the mutually exclusive expression of Ig domain proteins, notably *dumbfounded* (*duf*)*/kirre* in FCs and *sticks and stones* (*sns*) in FCMs [2–4]. Thus, *Drosophila* muscle development involves the mandatory asymmetric fusion of two cell types that display intrinsically different gene expression programmes. Alongside myoblast fusion, each prospective muscle elongates towards epidermal tendon cells to establish stable junctions with the exoskeleton at specific positions, after which myofibres become contractile [5]. This requires assembly of generic core sarcomeric proteins such as Myosin heavy chain (Mhc) [6].

The specific morphology of each muscle reflects the expression of a specific combination of identity transcription factors (iTFs) such as Apterous, Even-Skipped and Slouch/S59 by its FC [7, 8]. FCs originate from asymmetric, terminal division of progenitor cells (PCs) that are selected from equivalence groups of myoblasts called promuscular clusters (PMCs), while unselected myoblasts become FCMs [9, 10]. Specification of the FC iTF code integrates positional, temporal and homeotic information as well as extensive cross-regulations between different iTF genes at the PC and FC stages [11, 12]. By contrast, all FCMs express the Lameduck (Lmd) and Tramtrack Ttk69 transcription factors [13–15]. Yet, FCMs are derived from a PMC that transiently expresses specific iTFs [10, 16]. Whether this transient iTF expression has an impact on FCM fate is a long-standing question.

Whereas the FC/FCM fusion process has been decrypted in detail, the fate of syncytial FCM nuclei has not received the same attention. Expression studies on the S59 and Collier (Col) iTFs showed that FCM nuclei activate iTF gene transcription after fusion, revealing a conversion of syncytial nuclei towards FC-specific identity [17, 18]. The kinetics and selectivity of this conversion, and whether all nuclei in a syncytium behave in the same manner, remain to be established. One current hypothesis is that the FC iTF code controls fibre-specific expression of ‘realisation’ genes responsible for the stereotyped morphology of each muscle [11]. How the activation of realisation genes and that of generic differentiation genes common to all body wall muscles articulate with each other also remains unresolved.

Here, we addressed these different questions by studying the dynamics of transcription of three muscle iTF genes (*col*, *S59* and *Krüppel* (*Kr*) [19]), one FCM-generic gene (*sns*), one FC-generic gene (*duf*), the generic muscle differentiation gene (*Mhc*) [20], and four putative realisation genes (*Connectin* (*Con*) [21, 22], *kon*-*tiki/perdido* (*kon*) [23, 24], *M-spondin* (*mspo*) and *Paxillin* (*Pax*) [25–27]), in a group of dorsolateral muscles. Using FISH with intronic probes to detect nascent transcripts, we describe the temporal window(s) of transcription of each gene in individual nuclei within different syncytia. Statistical analyses take into account the variations in signal due to the discontinuous character of transcription, which is comprised of a succession of transcriptional bursts, followed by periods of little or no transcription [28, 29]. We could thus compare the transcriptional status of different genes in individual nuclei, within and between syncytia.

Our data show that FCMs from different PMCs can be incorporated into a given myofibre and are equally competent to be converted to a specific muscle identity. Transcriptional re-programming of FCM nuclei is sequential, with the loss of FCM-specific transcription preceding activation of FC genes. Fibre-specific expression of realisation genes is only activated in nuclei transcribing iTFs, and is regulated at both the level of transcription and the period of transcriptional activity. Finally, we show that the muscle generic and identity transcription programmes are regulated independently. Our results provide a novel view of the dynamics of transcriptional reprogramming of post-mitotic nuclei within a syncytium, and a new framework for understanding the transcriptional control of muscle identity, a fundamental process in the animal kingdom.

## Results

### FCMs are naive myoblasts

**Fig. 1:**
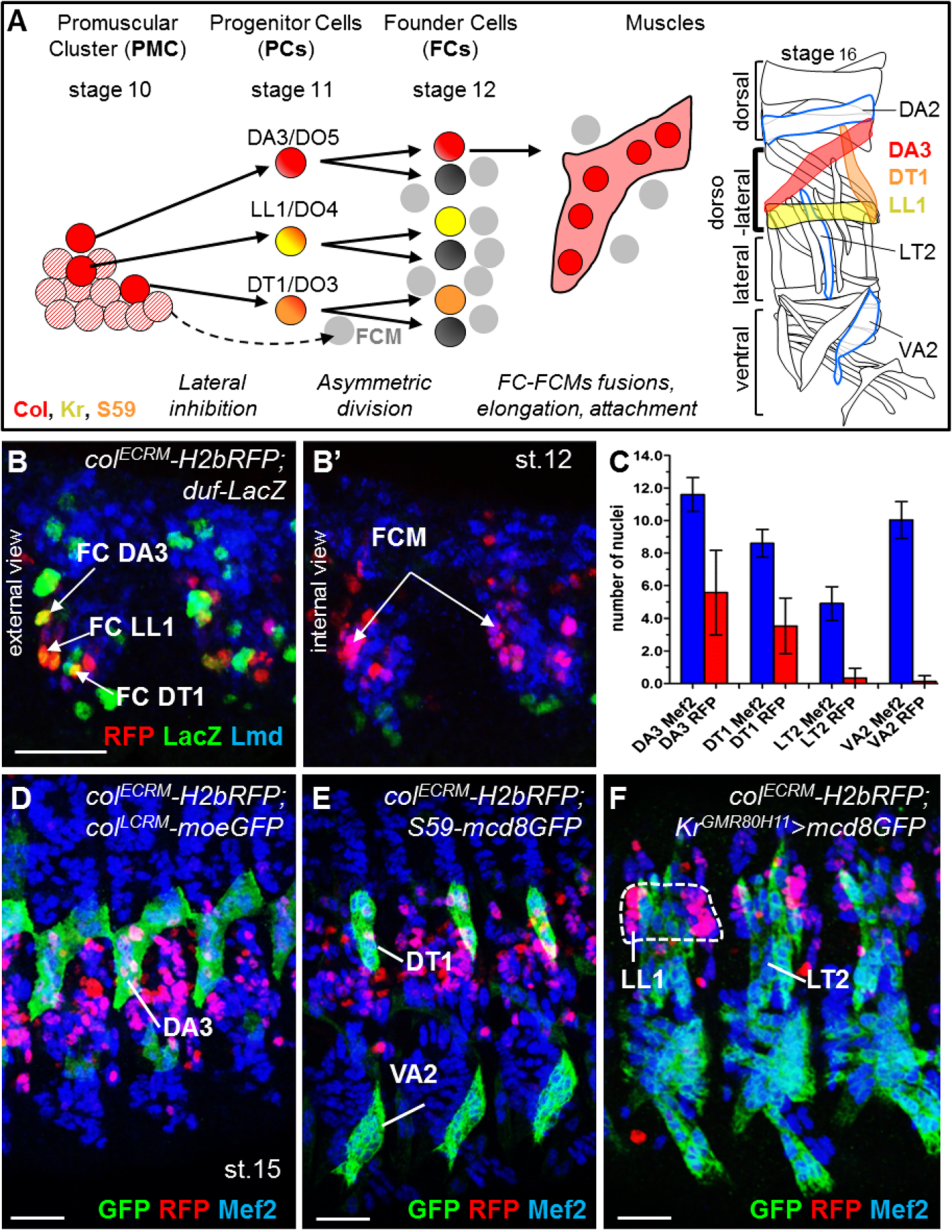
Tracking fusion competent myoblasts (FCMs) derived from the Col^+^ promuscular cluster. *A* Schematic representation of dorso-lateral muscle formation, with embryonic stages indicated. The DA3/DO5, LL1/DO4 and DT1/DO3 progenitor cells (PCs) are selected from a promuscular cluster expressing Col (*red hatched*); unselected myoblasts become FCMs (*grey*). Each PC generates two founder cells (FCs) that fuse with FCMs to form syncytial fibres, which attach to tendon cells to form contractile muscles. The LL1 and DT1 FCs express Kr and S59, respectively (*colour coded*). Col expression is maintained in the DA3 muscle (*red*). Out-group dorsal DA2, lateral LT2 and ventral VA2 muscles are circled in *blue*, other muscles in *black*. *B*–*B’* Stage 12 *col* ^*ECRM*^ *-H2bRFP; duf-LacZ* embryo stained for RFP (*red*) to identify the Col^+^ promuscular cluster (PMC) nuclei, LacZ (*green*) and Lmd (*blue*) to visualise all FCs and all FCMs, respectively. *B* External and (*B’*) internal layers where FCs and FCMs are located, respectively; *C*–*F* Repartition of RFP^+^ FCM nuclei at stage 15. Box plots indicate the numbers of RFP^+^ relative to Mef2^+^ nuclei in DA3, DT1, LT2 and VA2 (*C*); *col* ^*ECRM*^ *-H2bRFP; col* ^*LCRM*^ *-moeGFP* (*D*), *col* ^*ECRM*^ *-H2bRFP; S59-mcd8GFP* (*E*) and *col* ^*ECRM*^ *-H2bRFP; Kr* ^*GMR80H11*^ *-Gal4,UAS-mcd8GFP* (*F*) embryos stained for RFP (*red*) and GFP (*green*) to outline the DA3 (*D*), DT1 and VA2 (*E*), and LT2 and LL1 (*F*) contours; Mef2 staining (*blue*) visualises all myoblast nuclei. Lateral views of embryos, dorsal up, anterior left; two adjacent abdominal hemisegments in B, B’, three in D-F; scale bar: 20 μm. In C, bar graphs indicate the mean, and error bars the SD

One key step in generating the stereotyped *Drosophila* skeletal muscle pattern is the specification of muscle PC and FC identity. PCs and FCMs both derive from equivalent groups of cells, called PMCs (Fig. 1A) [9, 10]. All myoblasts in a given cluster express some iTFs in response to positional information. iTF expression is maintained in PCs and some FCs, while lost in FCMs (Fig. 1A) [16]. Hence, a long-standing question in the field is whether transient activation of specific iTF(s) in prospective FCMs biases their fate?

**Fig. 2:**
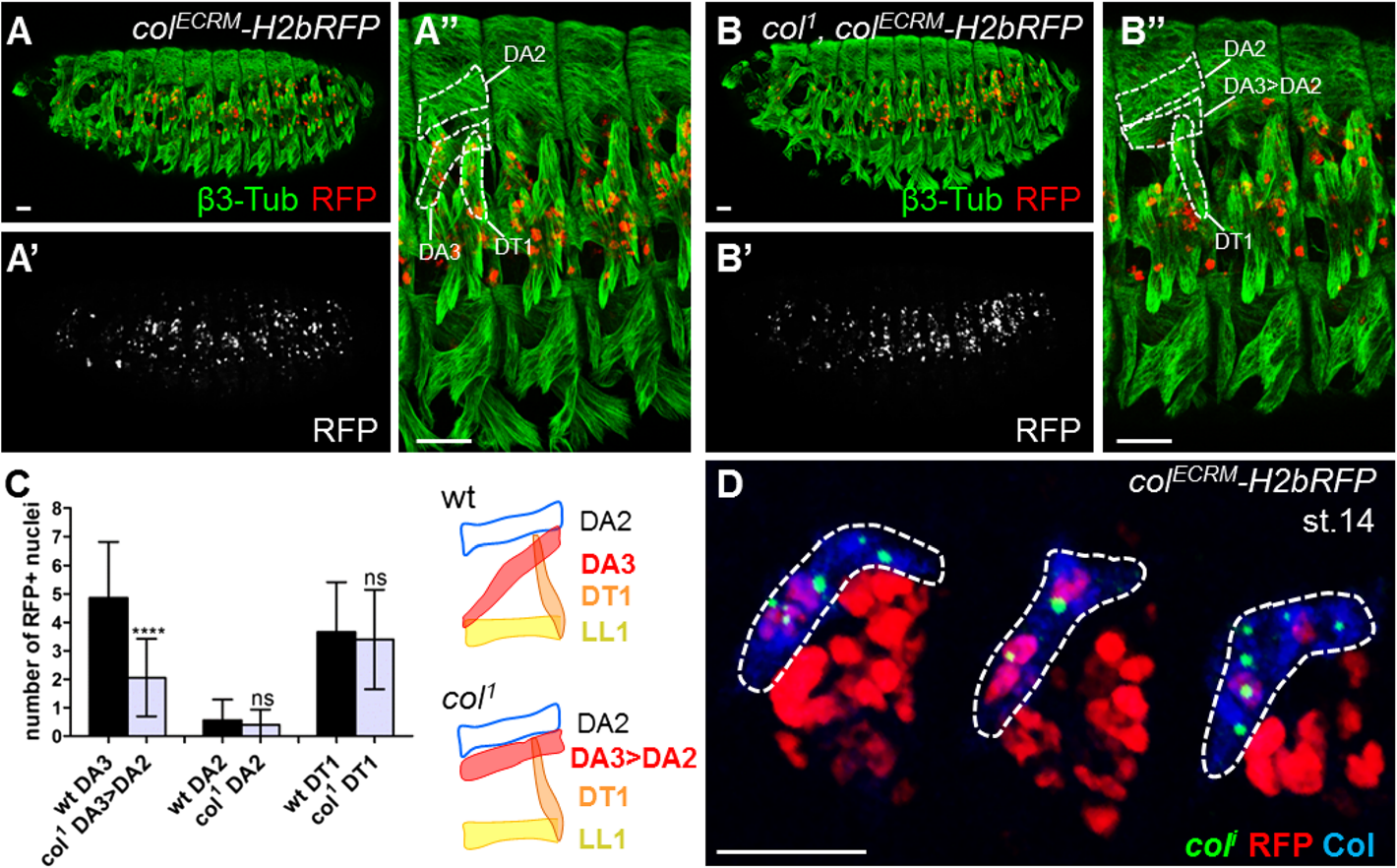
Promuscular *col* expression does not impact fusion competent myoblast (FCM) fate. Stage 15 *col* ^*ECRM*^ *-H2B-RFP* (*A*) and *col* ^*1*^ *,col* ^*ECRM*^ *-H2B-RFP* (*B*) embryos stained for RFP (*red*) and β3-tub (*green*) to visualise the nuclei of Col^+^ PMC myoblasts and all muscles, respectively; (*A’*, *B’*) red channel only; (*A”*, *B”*) close up of three abdominal hemisegments. *C* Number of RFP^+^ nuclei in DA2, DA3 and DT1 at stage 15 in *col* ^*ECRM*^ *-H2bRFP* and DA2, DA3 > DA2 and DT1 in *col* ^*1*^ *,col* ^*ECRM*^ *-H2bRFP* embryos. The DA3 transformation into a DA2-like muscle (DA3 > DA2) in *col* ^*1*^ embryos is schematised on the right. *D* FISH of nascent *col* transcripts (*green*) in stage 14 *col* ^*ECRM*^ *-H2bRFP* embryos stained for RFP (*red*) and Col (*blue*); *col* is transcribed in a fraction of both RFP^+^ and RFP^–^ DA3 nuclei. Scale bar: 20 μm

In order to explore this question, we used as a paradigm the Col-expressing PMC from which the DA3/DO5, DT1/DO3 and LL1/DO4 PCs are selected, after which only the DA3 FC and muscle maintain Col expression (Fig. 1A) [18, 30]. In order to specifically label Col-expressing PMC myoblasts and follow their fate, we expressed H2B-RFP under the control of the PMC *col* cis-regulatory module (CRM), *col* ^*ECRM*^ (previously called CRM276 [31]). LacZ expression under the control of cis-regulatory elements of the FC-specific gene *duf* (duf-LacZ) and Lmd served to visualise all FCs and FCMs, respectively, at stage 12. As expected, H2B-RFP staining was detected in nuclei of FCs and FCMs (Fig. 1B, B’). Due to its stability and nuclear retention, H2B-RFP expression allowed tracking of the repartition of myoblasts derived from the Col PMC in developing myofibres up to stage 15. It showed that RFP^+^ nuclei were mainly incorporated in dorsolateral muscles and occasionally found in more dorsal as well as ventral-lateral muscles. This repartition suggests that FCM repartition follows neighbouring cues. We precisely quantified the fraction of RFP^+^ nuclei within DA3 and DT1, each of the four lateral transverse (LT) muscles, and the ventral VA2 muscle (Fig. 1 and Additional file 1: Table S1). For this quantification, all myoblast nuclei were visualised by Mef2 staining (Fig. 1C–F). The DA3 and DT1 plus VA2 contours were identified by *col* ^*LCRM*^- (late *col* CRM) and *S59*-driven GFP expression, respectively (Fig. 1D, E), and the contours of LTs by mCD8-GFP expression under control of a *Kr* muscle CRM (Fig. 1F), which we identified by systematically testing *Kr*-linked GMR lines (Additional file 2: Figure S1 and [32]). In either DA3 or DT1 dorsolateral muscles, approximately 50% of nuclei were RFP^+^ (Fig. 1C and Additional file 1: Table S1). A similar proportion was observed in LL1, a third dorsolateral muscle (Fig. 1F). In contrast, both lateral LTs, or ventral VA2 muscles contained few RFP^+^ nuclei (Fig. 1C, E, F and Additional file 1: Table S1), supporting the conclusion that FCM recruitment follows local cues. Recruitment of both RFP^+^ and RFP^–^ FCMs into DA3 and DT1 further shows that FCMs are not pre-determined to contribute to a given muscle by early iTF expression. To further strengthen this conclusion, we analysed the repartition of H2B-RFP nuclei in *col* ^*1*^ mutant embryos expressing *col* ^*ECRM*^ *-H2B-RFP*, with *col* ^*ECRM*^ activity being independent of Col. In *col* ^*1*^ embryos, the DA3 muscle is transformed into a more dorsal, DA2-like muscle (DA3 > DA2), while DT1 morphology is unchanged [30] (Fig. 2A, B). We found that the average number of RFP^+^ nuclei incorporated into DT1 was unchanged (~3.5; Fig. 2C and Additional file 3: Table S2). However, it was lower in the dorsalised DA3 > DA2 muscle than in DA3 (~2, compared to ~5; Fig. 2C and Additional file 3: Table S2), while remaining higher than in dorsal DA2 (~0.5; Fig. 2C and Additional file 3: Table S2). These data show that the recruitment of FCMs in distinct muscles during the elongation process follows topological rules.

Finally, we investigated whether fused FCMs were differentially competent to transcribe FC iTF(s), depending upon their PMC origin by analysing *col* transcription in DA3 muscles of *col* ^*ECRM*^ *-H2B-RFP* embryos, which contain a mixture of RFP^+^ and RFP^–^ nuclei. We found that both RFP^+^ and RFP^–^ nuclei activate *col* transcription (Fig. 2D), showing that FCM nuclei can be reprogrammed to a given identity, independent of prior iTF expression.

### iTF gene transcription in syncytial nuclei is both transient and iTF-specific

**Fig. 3:**
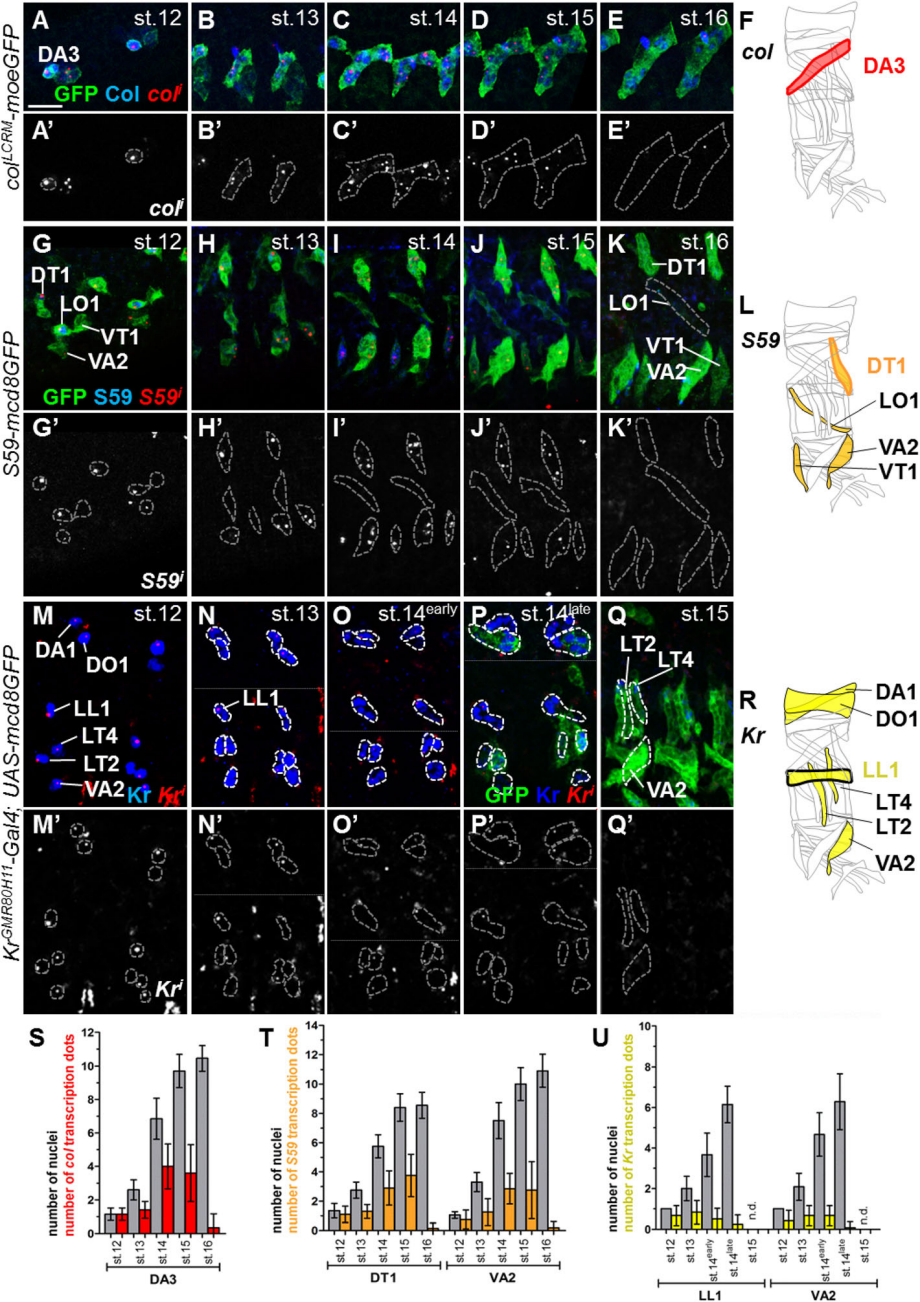
Dynamics of identity transcription factor transcription during muscle development. (*A*–*E*’) *col* ^*LCRM*^ *-moeGFP*, (*G*–*K’*) *S59-mcd8GFP* and (*M*–*Q’*) *Kr* ^*GMR80H11*^ *-Gal4;UAS-mcd8GFP* embryos stained for nascent *col* (*A*–*E*’), *S59* (*G*–*K’*) or *Kr* (*M*–*Q’*) transcripts (*red*) and GFP (*green*) to visualise muscle shape, and either Col (*A*–*E’*), S59 (*G*–*K’*) or Kr (*M*–*Q’*) (*blue*). (*A’*–*E’*, *G’*–*K’*, *M’*–*Q’*) *red* channel only, with dotted grey lines outlining muscle shape. *Kr* is transcribed in a single nucleus from stage 12 to late stage 14, in all Kr-expressing muscles. (*F*, *L*, *R*) Schematic view of the Col^+^, S59^+^ and Kr^+^ muscles *colour coded* as in Fig. 1A. (*S*–*U*) *Box plots* showing the relative numbers of nuclei and *col* transcription dots in DA3 (*S*), *S59* dots in DT1 and VA2 (*T*), and *Kr* dots in LL1 and VA2 (*U*)

Although transcriptional conversion of FCM nuclei to FC identity upon fusion is often presented as a dogma, it has only been documented for *col* in the DA3 and *S59* in the DT1, VA2 and VT1 muscles [17, 18]. To determine the extent and dynamics of transcriptional conversion in different muscles, we reinvestigated the patterns of *col* and *S59* transcription, using *col*- and *S59*-driven GFP expression to follow muscle development, step by step (Fig. 3). We also investigated the transcription pattern of a third iTF gene, *Kr*, in several muscles including dorsolateral LL1 (Fig. 1A and Fig. 3). Detection of nascent transcripts was followed by quantification of two parameters, namely the number of hybridisation dots (nuclei in a transcriptionally active phase) in the syncytium and the intensity of each transcription dot, as a proxy of the transcription initiation rate.

Simultaneous staining for Col, nascent *col* transcripts and *col* ^*LCRM*^ *-moeGFP* confirmed *col* active transcription in the DA3 FC and several syncytial nuclei within growing DA3 fibres (Fig. 3A–F and Additional file 4: Table S3), and stop of transcription at stage 16. Quantification of the nuclei number transcribing *col*, relative to the total number of DA3 syncytial nuclei, shows, however, that only a fraction transcribe *col* at a given time (Fig. 3S and Additional file 4: Table S3), suggesting transient transcription in each nucleus. The observation of growing DA3 fibres, in which *col* is transcribed in virtually all nuclei (Additional file 5: Figure S2A) shows that all fused FCM nuclei are competent to be converted to the FC identity transcriptional programme. The variable numbers of transcriptionally active nuclei in adjacent hemisegments (Additional file 5: Figure S2A) indicate that activation of *col* transcription in syncytial nuclei is stochastic rather than synchronous. Quantification of the intensity of *col* transcription dots shows that *col* is transcribed at a 9-fold higher level in the FC than in syncytial DA3 nuclei at stage 14 (Additional file 5: Figure S2B; Additional file 6: Table S4). Thus, two different modes of *col* regulation operate successively. This correlates with the previous identification of two distinct *col* mesodermal CRMs that act sequentially [31]. As for *col* in DA3, only a fraction of nuclei within DT1, VA2 or VT1 syncytia transcribe *S59* at a given time (Fig. 3G–L, T and Additional file 5: Figure S2C; Additional file 4: Table S3). Interestingly, this fraction differs between muscles, at approximately 45%, 27% and 23%, in DT1, VA2 and VT1, respectively, at stage 15 (Additional file 5: Figure S2D), while the integrated density of *S59* dots is relatively constant from stage 12 to 15 (Additional file 5: Figure S2E; Additional file 6: Table S4). Together, these data suggest that the time interval of *S59* transcription is differentially regulated in DT1, VA2 and VT1. S59 is also expressed in LO1 at stage 12, but in this lineage, *S59* is only transcribed in the FC at stage 12 and not at later stages (Fig. 3G–L and Additional file 5: Figure S2C; Additional file 4: Table S3) [17]. Finally, FISH of nascent transcripts shows that *Kr* transcription in Kr^+^ muscles ceases at early stage 14 (Fig. 3M–R). Unexpectedly, *Kr* transcription is detected at a constant level in a single nucleus per fibre, from stage 12 to 14 (Fig. 3M–R, U and Additional file 5: Figure S2F, G; Additional files 7 and 8: Tables S5 and S6). The *Kr* ^*+*^ nucleus stays at roughly the same position within the muscle throughout the process of fibre growth, suggesting that it is the FC nucleus and that *Kr* transcription is maintained in the FC nucleus during the fusion process without being propagated to other syncytial nuclei. Up to now, two ‘typical’ iTF gene transcription patterns have been reported, namely repression at the PC or FC stage (for example, *col* in DT1 and LL1, *S59* in DA3 [12]) or, alternatively, maintenance and propagation of transcription to other syncytial nuclei (for example, *col* in DA3, *S59* in DT1). *Kr* transcription in a single nucleus up to stage 14 reveals a novel pattern of iTF transcriptional regulation in muscle precursors, namely maintenance in the FC nucleus without propagation to FCM nuclei.

Together, the observed patterns of nascent *col*, *Kr* and *S59* transcripts show that activation of iTF transcription in FCMs after fusion is iTF- and muscle-specific, and cannot be considered as a general rule. Furthermore, single nucleus *Kr* transcription provides the first evidence that the FC nucleus could maintain a transcriptional programme different from the other syncytial nuclei during muscle development.

### Transcriptional conversion of FCMs to syncytial identity

Fusion of FCMs with an FC and, subsequently, the derived growing fibre, is based on mutually exclusive expression of the surface proteins Duf/Rst and Sns by all FCs and FCMs, respectively [33]. For this to be a reiterative process implies that, upon fusion, FCM nuclei switch off FCM-specific gene transcription (*sns*) and that the FC-generic *duf* gene remains expressed. This process remains to be decrypted at the transcriptional level.

**Fig. 4:**
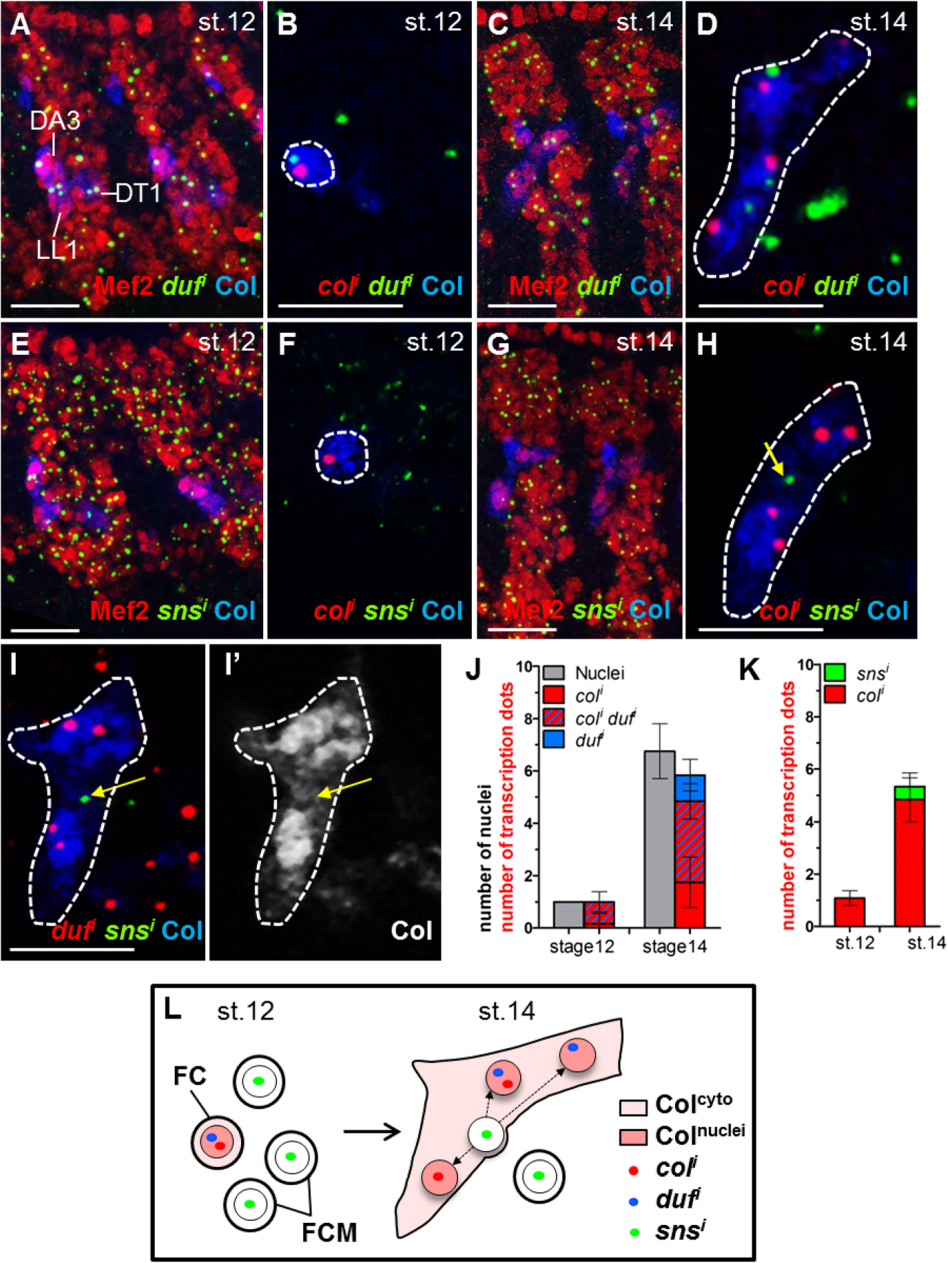
Transcriptional conversion of fused fusion competent myoblast (FCM) nuclei to founder cell (FC) identity. FISH against nascent *duf* (*A*, *C*) or *sns* (*E*, *G*) transcripts (*green*), staining for Mef2 (*red*), and Col (*blue*) to visualise the DA3, DT1 and LL1 FC nuclei at stage 12 (*A*, *E*) and the DA3 muscle at stage 14 (*C*, *G*), wt embryos. Double FISH against nascent *col* (*red*) and either *duf* (*B*, *D*) or *sns* (*F*, *H*) transcripts (*green*) and Col staining (*blue*) of the DA3 FC at stage 12 (*B*, *F*) and DA3 muscle at stage 14 (*D*, *H*). (*I*, *I’*) Double FISH against nascent *duf* (*red*) and *sns* transcripts (*green*) and Col staining, stage 14 DA3 muscle; (*I’*) *blue* channel only. (*H*, *I*, *I’*) *sns* transcription (*yellow arrow*) is detected in "no Col" nuclei. (*J*, *K*) Box plots showing the number of nuclei transcribing either *col* or *duf*, or *col* and *duf*, relative to the total number of DA3 nuclei (*J*), and either *col* or *sns* only (*K*). Two adjacent hemisegments are shown in (*A*, *C*, *E*, *G*), section projections; a single hemisegment in (*B*, *D*, *F*, *H*, *I*), single confocal sections. *L* Schematic representation of the transcriptional FCM to generic FC identity switch, post fusion

We sought to determine precisely when the FCM to FC/fibre transcription switch occurs, using DA3 as a paradigm. FISH to *duf* nascent transcripts confirmed that *duf* is transcribed in all FCs at stage 12, including the DA3, DT1 and LL1 FCs labelled with Col (Fig. 4A; Fig. 1A) [30]. At stage 14, the increased number of *duf* transcribing nuclei in DA3 syncytia correlates with the increased number of recruited FCM nuclei (Fig. 4C, J), demonstrating activation of the FC-generic transcriptional programme in FCM nuclei. *duf* transcription drops at late stage 15, at the end of the fusion process [27, 34] (Additional file 9: Figure S3A–C). Paired FISH of nascent *duf* and *col* transcripts shows that the DA3 FC co-transcribes *duf* and *col* at stage 12 (Fig. 4B). However, at stage 14, while 80% of syncytial nuclei transcribe at least one of these two genes, only 50% of these nuclei co-transcribe *duf* and *col* (Fig. 4D, J and Additional file 9: Figure S3D, E; Additional file 10: Table S7), possibly reflecting asynchronous bursts of *col* and *duf* transcription. Their dynamics of transcription show that *duf* and *col* activation is not synchronised and independent of each other in individual nuclei within a syncytium. We previously showed that nuclear uptake of Col protein precedes activation of *col* transcription in fused FCM nuclei [35]. The detection of *duf* dots in nuclei with very low levels of Col protein (Additional file 9: Figure S3D) further supports independent activation of *duf* and *col*.

Conversely, nascent *sns* transcripts are detected in a large number of FCMs surroundings FCs at stage 12, but not in FCs themselves (Fig. 4E, F, K and Additional file 10: Table S7). During the fusion period, only one, if any, *sns* transcription dot is detected in growing DA3 myotubes (Fig. 4H, K and Additional file 9: Figure S3 E; Additional file 10: Table S7), indicating that switching off the FCM transcriptional programme occurs post fusion. Furthermore, no nuclei co-transcribing *sns* and *col* (Fig. 4H, K and Additional file 10: Table S7), or *sns* and *duf* (Fig. 4I) were detected, indicating that repression of FCM-specific genes precedes activation of FC-generic and iTF transcription. In support of this, *sns* dots could only be detected in nuclei displaying no or very low levels of Col protein (Fig. 4I, I’; [35]).

In summary (Fig. 4L), the *duf*, *sns* and *col* transcription patterns show that transcriptional reprogramming of FCMs is a dynamic process: after fusion of FCMs, transcription of FCM-generic genes (*sns*) is first turned off, followed by a switch from FCM to FC-generic (*duf*) and FC-specific (*col*) transcriptional programmes. These latter two aspects of transcriptional activation are asynchronous.

### Transcription of generic muscle differentiation genes is independent of iTF gene transcription

**Fig. 5:**
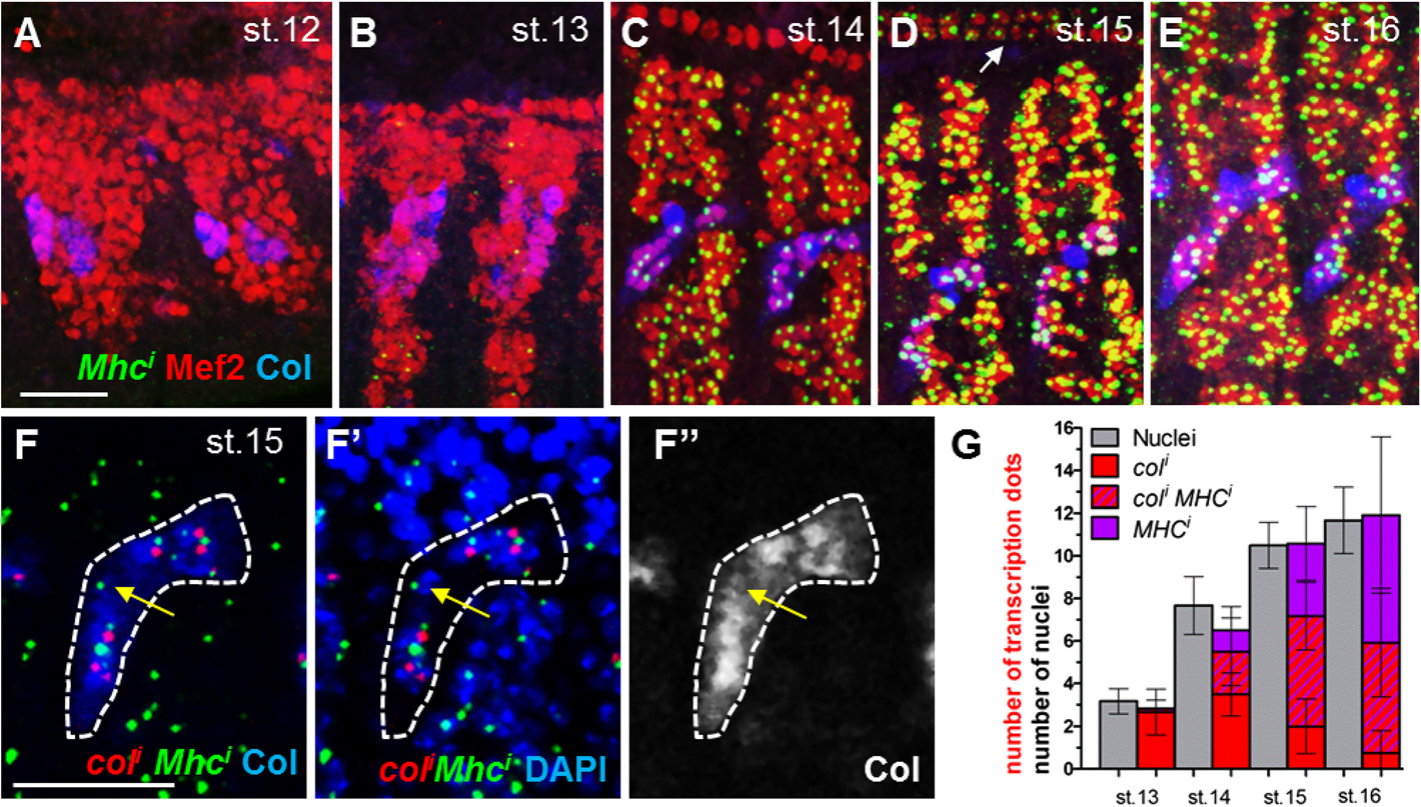
Independent transcription of identity transcription factors and generic muscle differentiation genes. *A–E* wt embryos at various embryonic stages, stained for Mef2 (*red*) and Col (*blue*) coupled to FISH against nascent *Mhc* transcripts. The white arrow points to *Mhc* expression in heart cells, first detected at stage 15. *F* Double FISH against nascent *col* (*red*) and *Mhc* (*green*) transcripts, and Col staining (*blue*) to visualise the DA3 nuclei at stage 15. *F’* same as (*F*), DAPI staining (*blue*) shows all nuclei. *F”* Col staining only. Single Z sections. The *yellow arrow* indicates a nucleus with low Col protein, which transcribes *Mhc*. *G* *Box plots* showing the number of nuclei transcribing either *col*, *Mhc* or both, relative to the total number of DA3 nuclei

Assembly of sarcomeres underlies the formation of contractile muscles. Consistent with Mhc being a critical sarcomeric component, *Mhc* mRNA level is similar in all muscle fibres at stage 15 (Additional file 11: Figure S4A). FISH of nascent transcripts indicated that *Mhc* transcription is first weakly detected in few nuclei at stage 13, and increases dramatically at stage 14, simultaneously in all developing muscles (Fig. 5A–D). The homogenous distribution of *Mhc* dots suggests that transcription of generic muscle differentiation is independent of muscle identity. Supporting this conclusion, high-level *Mhc* transcription is maintained in muscle syncytia until the end of embryogenesis, when iTF transcription has already strongly decreased (Fig. 5E and Fig. 3). To further assess whether the generic differentiation and muscle identity transcription programmes are coupled, we compared the dynamics of *col* and *Mhc* transcription in the developing DA3 muscle. Paired FISH of nascent transcripts, coupled to Col and DAPI staining (Fig. 5F–F”, G and Additional file 12: Table S8), confirmed that virtually all DA3 nuclei transcribe *Mhc* at stage 15, while only roughly a quarter also transcribes *col* at a given time point (Fig. 5G and Additional file 12: Table S8). Together, these data support the conclusions that the generic muscle differentiation and identity programmes are uncoupled temporally and independently activated in syncytial nuclei (Fig. 5).

### Fibre-specific transcription of identity realisation genes; multi-level regulation

The current hypothesis is that the iTF code specific to each FC controls the differential expression of genes underlying ‘realisation’ of muscle morphological identity. A search for candidate realisation genes identified two cytoskeletal protein genes, *Pax* and *mspo*, which display fibre-specific expression levels and are involved in fibre-specific modulation of the fusion rate [27]. Two other cell surface proteins, Con and Kon, were independently implicated in fibre-specific regulation of muscle attachment. *kon* encodes a transmembrane protein required in a specific subset of myotubes to recognise and establish stable connections with appropriate tendon cells [5, 23]. *kon* was also recovered in a screen for FC-specific genes based on transcriptomics and computational analyses under the name of *perdido* [24, 36]. *Con* encodes a cell surface protein decorating a subset of muscles, including DT1, and the motoneurons which innervate them [22, 37].

**Fig. 6:**
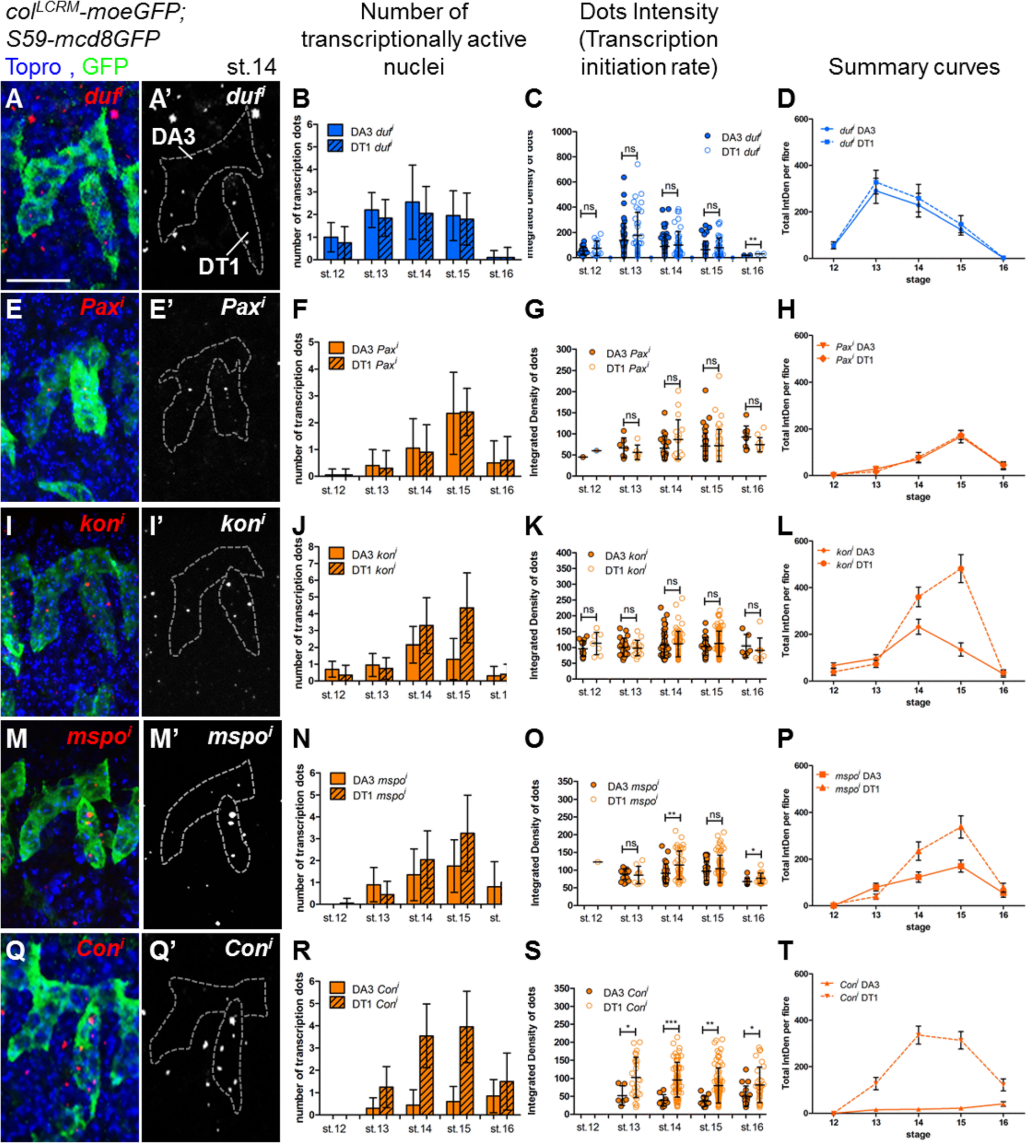
Transcription dynamics of identity-realisation genes. *A*, *E*, *I*, *M*, *Q* Stage 14 *col* ^*LCRM*^ *-moeGFP; S59-mcd8GFP* embryos stained for GFP (*green*) and Topro (*blue*) to visualise DA3 and DT1 and all nuclei, respectively, coupled with FISH of either nascent *duf* (*A*), *Pax* (*E*), *kon* (*I*), *mspo* (*M*) or *Con* (*Q*) transcripts (*red*); *A’*, *E’*, *I’*, *M’*, *Q’* Red channel only, muscle contours are outlined by *dashed grey lines*. *B*, *F*, *J*, *N*, *R* Number of transcription dots in DA3 and DT1 at different embryonic stages. Bar graphs indicate the mean number of nuclei; error bars, the SD. *C*, *G*, *K*, *O*, *S* Intensity (IntDen) of transcription dots in DA3 and DT1; each *dot* is represented by a *circle*, the *bar graphs* show the mean value and SD. *D*, *H*, *L*, *P*, *T* Cumulative intensity of transcriptional dots (Total IntDen per fibre) in DA3 and DT1. The mean value is shown; error bars correspond to the SEM

We compared the transcription patterns of *Pax*, *mspo*, *kon* and *Con* in DA3 and DT1, which present the advantage of displaying similar numbers of nuclei and FCM recruitment kinetics (Additional file 11: Figure S4B). FISH of mature transcripts at stage 15 showed that all four genes are expressed in both muscles. Yet, while *Pax* is expressed at similar levels in DA3 and DT1, *mspo*, *kon* and *Con* mRNAs accumulate at significantly higher level in DT1 (Additional file 11: Figure S4D, F, H, J; Additional file 13: Table S9). FISH of nascent transcripts in stage 14 embryos further suggested that differential accumulation of *mspo*, *kon* and *Con* mRNA reflects differential transcription (Additional file 11: Figure S4E, G, I, K). We therefore performed a side by side comparison of the dynamics of transcription of *Pax*, *mspo*, *kon* and *Con* between DA3 and DT1, by both measuring the numbers of hybridisation dots and individual dot intensities for each gene in syncytia, at embryonic stages 12, 13, 14, 15 and 16, using *duf* as internal reference (Fig. 6 and Additional files 14 and 15: Tables S10 and S11).

As reported above, the number of *duf* dots increases between stages 12 and 14, in both DA3 and DT1 (Fig. 4J, Fig. 6B and Additional file 14: Table S10) and dot intensity is maximal during the muscle elongation phase, stages 13 to 15, both in DA3 and DT1 (Fig. 6C and Additional file 15: Table S11). The numbers and intensity of *Pax* transcription dots are also similar in DA3 and DT1, during the fusion phase (Fig. 6F, G). A maximum of 2.5 dots per fibre at stage 15 suggests that transcription is stochastic and/or transient in syncytial nuclei. The absence of dot detection in FCs at stage 12 indicates that *Pax* transcription is activated post fusion. Contrary to *Pax*, *kon* transcription is detected in the DA3 and DT1 FCs, and the kinetics of *kon* transcription differs between DA3 and DT1. The number of *kon* dots increases between stages 14 and 15 in DT1, reaching an average of 4.35 per fibre, while it decreases earlier in DA3 (Fig. 6J and Additional file 14: Table S10). Nevertheless, dot intensity indicates that the *kon* transcription initiation rate is not significantly different between the two muscles (Fig. 6K and Additional file 15: Table S11). Together, these data indicate that differential transcription of *kon* between DA3 and DT1 reflects different time intervals of transcription per nucleus. However, a third scenario is observed for *mspo*, with higher dot number and intensity in DT1 than in DA3 (Fig. 6N, O and Additional files 14 and 15: Tables S10 and S11). Thus, both differences in the time of transcription and transcription initiation rate can contribute to differential *mspo* transcription in different muscles during the acute fusion phase. This pattern is even more pronounced for *Con*, where high numbers of high intensity dots are detected in DT1, correlating with Con accumulation at the surface of the DT1 muscle [22], and rare, low intensity dots are detected in DA3 (Fig. 6Q–S and Additional files 14 and 15: Tables S10 and S11). Summary curves corresponding to the mean sum of all dot intensities measured in each fibre underline the differential transcription of *kon*, *mspo* and *Con* between DA3 and DT1 (Fig. 6D, H, L, P, T and Additional file 16: Table S12). Furthermore, they show that the transcription level of realisation genes is regulated in a fibre-specific manner, via the modulation of the transcription initiation rate and/or time interval of active transcription per syncytial nucleus.

### Transcriptional activation of realisation genes follows iTF gene activation

**Fig. 7:**
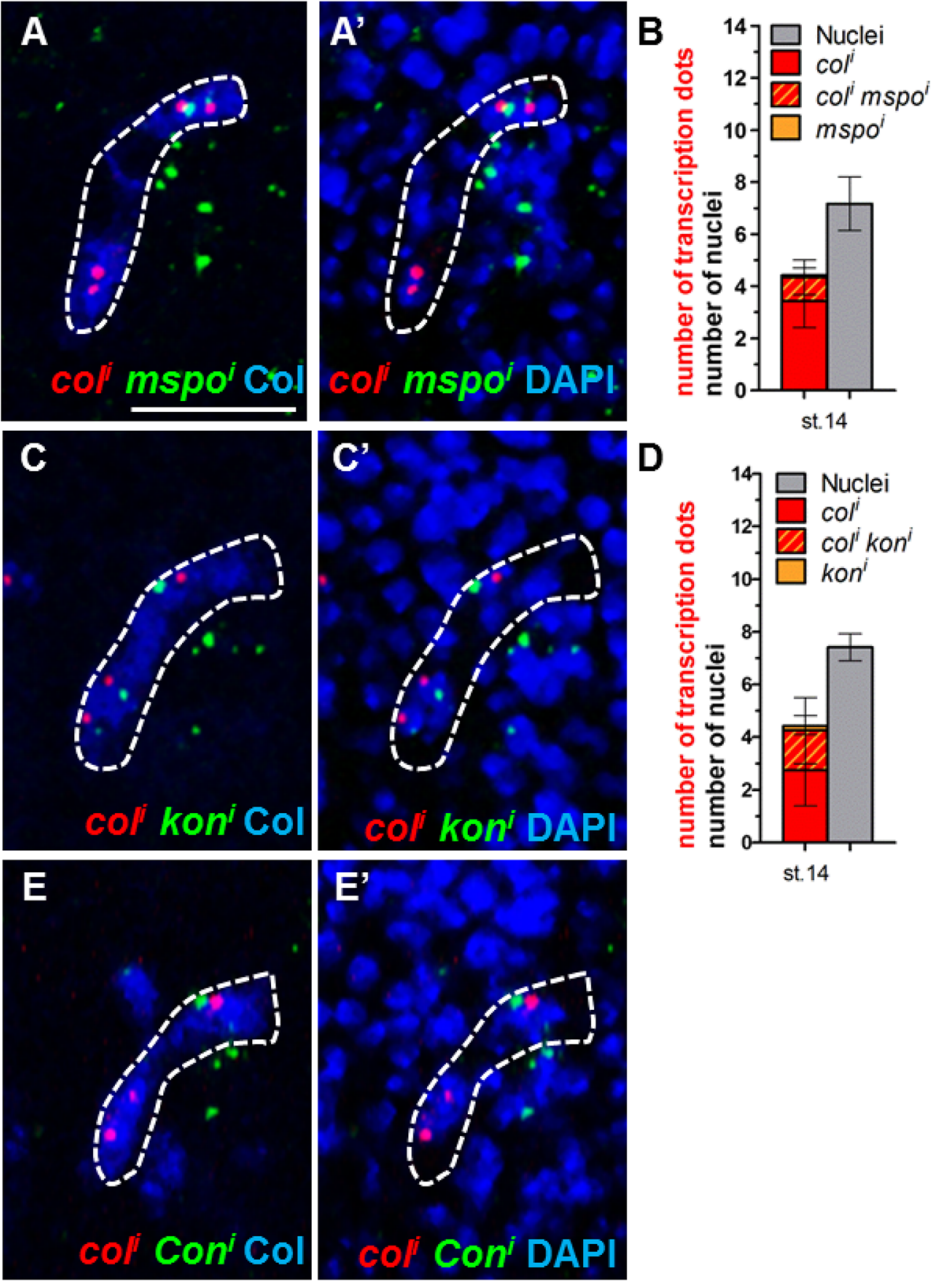
Transcription of identity-realisation genes is coupled to identity transcription factor transcription. Double FISH of nascent *col* (*red*) and either *mspo* (*A*), *kon* (*C*) or *Con* (*E*) (*green*) transcripts, Col staining for (*blue*) visualising the DA3 nuclei at stage 14. *A’*, *C’*, *E’* DAPI staining (*blue*) of all nuclei; single Z sections. *Box plots* showing the number of nuclei transcribing either *col*, *mspo* or both (*B*), and either *col*, *kon* or both (*D*), relative to the total number of DA3 nuclei

To determine whether transcription of identity realisation genes is connected to iTF activation, we focused on *mspo*, *kon* and *Con*, which are differentially expressed in DA3 versus DT1. Double FISH of *col* and either *mspo*, *kon* or *Con* nascent transcripts allowed us to measure the number of nuclei, either transcribing one or both genes, in developing DA3 (Fig. 7 and Additional file 12: Table S8). The numbers show that *mspo* or *kon* are only transcribed in nuclei also transcribing *col*. Thus, contrary to the asynchronous transcription of iTFs with respect to the FC-generic gene *duf* (Fig. 4), transcription of realisation genes is linked to iTF transcription in syncytial nuclei.

### The FC nucleus remains distinct from the other syncytial nuclei

**Fig. 8:**
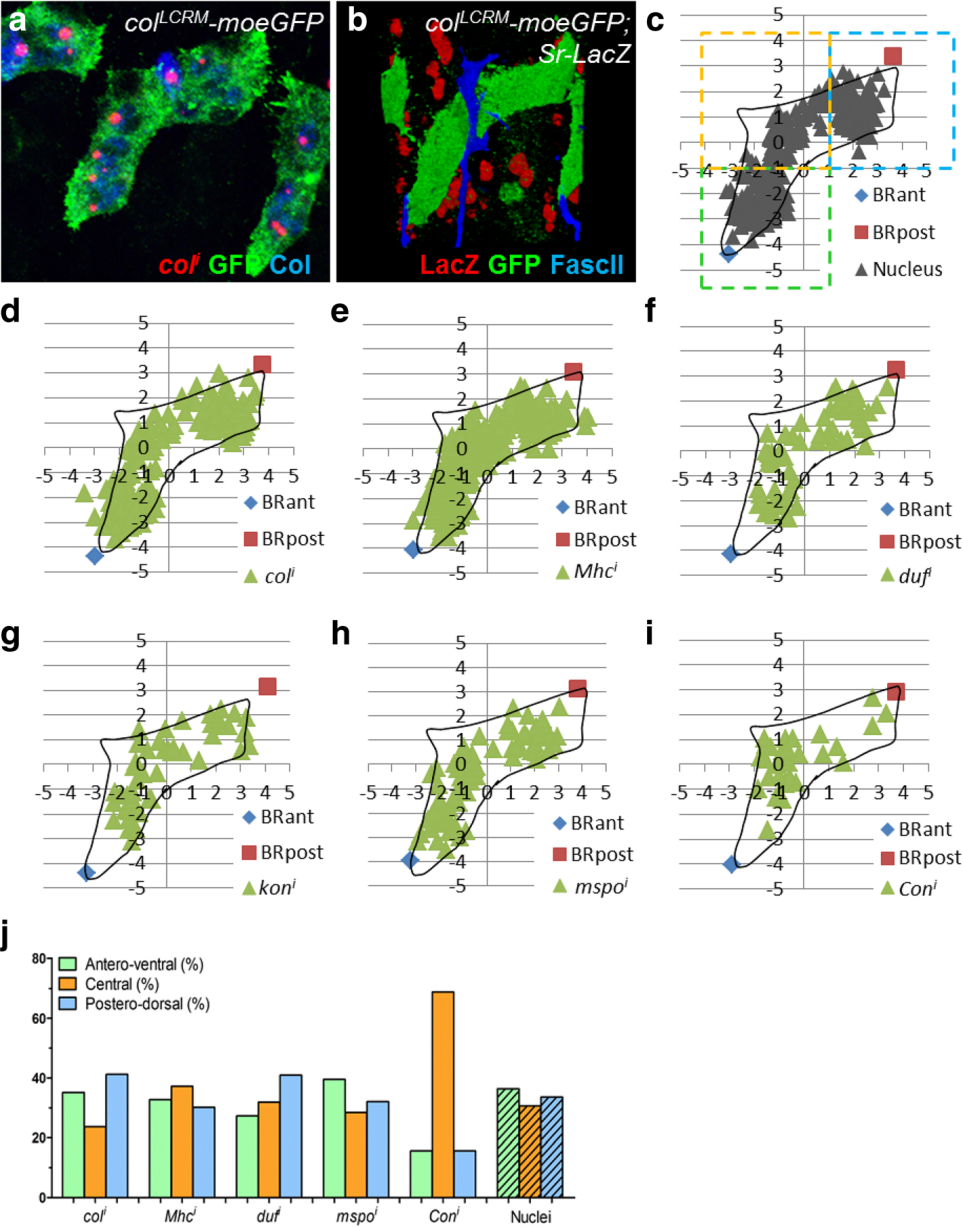
Transcription patterns and DA3 muscle subdomains. **a**, **b** Late stage 14 *col* ^*LCRM*^ *-moeGFP* embryo stained for GFP (*green*) and either Col (*blue*), coupled with FISH of nascent *col* transcripts (*red*) (**a**), or LacZ and Fasciclin II (FascII) to visualise tendon cells and the DA3 innervating motoneuron, respectively. **c** Subdivision of the DA3 muscle into ventral, median and dorsal subdomains shows an homogeneous distribution of nuclei; BRant and BRpost indicate the anterior and posterior DA3 limits, respectively (Additional file 17: Figure S5). Spatial distribution of *col* (**d**), *Mhc* (**e**), *duf* (**f**), *kon* (**g**), *mspo* (**h**) and *Con* (**i**) transcription dots. **j** Repartition of hybridisation dots and nuclei (hatched bar) in each DA3 subdomain

The variable numbers of syncytial nuclei transcribing different realisation genes raised the additional question of whether functional specialisation of nuclei could exist, according to their position within the syncytial myotube. At late stage 14, the DA3 muscle displays an angled shape, reflecting transient tripartite attachment to tendon cells (Fig. 8a, b); the final acute orientation of DA3 corresponds to stabilisation of the ventral-most anterior, and the dorsal-posterior attachment sites, which critically depends upon appropriate levels of Col and Nau activity [30]. The DA3 branch of the intrasegmental nerve contacts the ventral side of the angled DA3 muscle at a stereotypic position, roughly at its centre [38] (Fig. 8b). The position of the neuromuscular junction and the tripartite attachment sites provide morphological landmarks allowing the division of the DA3 muscle into three subdomains, namely antero-ventral, central and postero-dorsal (Fig. 8c–j). We analysed the topological distributions of nuclei and transcription dots for muscle generic (*Mhc*), FC-generic (*duf*) and identity genes (*col*, *kon*, *mspo* and *Con*), relative to these three subdomains. Plotting the spatial coordinates of each FISH dot and nucleus, relative to the DA3 shape (see Methods and Additional file 17: Figure S5), showed a roughly homogenous distribution of nuclei and *col*, *duf*, *kon*, *Mhc*, *mspo* and *Pax* transcriptional dots (Fig. 8c–h, j and Additional file 18: Table S13). Thus, transcriptional activation of these genes does not appear to be influenced by nucleus position. This could be required for homogeneous distribution of transcripts coding for surface proteins during muscle growth.

Most *Con* transcription dots (close to 70%; Fig. 8i, j and Additional file 18: Table S13) map to the central subdomain of the DA3 muscle. Interestingly, *Con* is the only realisation gene in our study which is, on average, transcribed in a single nucleus throughout DA3 development, suggesting that this is the FC nucleus, in agreement with the observation that Con is expressed in FCs [39]. Taken together, single nucleus-restricted *Kr* (Fig. 3) and *Con* transcription support the conclusion that the FC nucleus remains located at a stereotypical position and is transcriptionally different from the other syncytial nuclei throughout muscle development.

## Discussion

In its simplest terms, myogenesis is the differentiation of individual precursor cells, the myoblasts, into syncytial contractile myofibres. Yet, each body wall muscle displays a morphological identity. Understanding how transcriptional regulation of a generic myogenic programme, common to all muscles, and each muscle identity programme are integrated to generate morphological muscle diversity remains a central question in the field. Here, we used *Drosophila* larval muscles to address this question, exploiting hybridisation to nascent transcripts to compare the transcriptional status of a selection of generic and identity genes, in individual nuclei within different syncytial muscles. Our data show that transcriptional reprogramming of fused myoblast nuclei is progressive. Transcriptional activation of identity realisation genes is specific to nuclei expressing iTF genes and subject to different modes of regulation in a fibre-specific manner. Conversely, transcription of generic muscle differentiation genes is regulated independently of muscle morphological identity.

In humans, in addition to muscles, there exist two other types of differentiating multinucleated syncytia, the osteoclasts and syncytial trophoblasts. The transcriptional status of each nucleus in the different types of multinucleated cells and how it contributes to lineage diversity remains a widely open, fundamental field of investigation.

### Muscle ‘founder’ and naive myoblasts

The development of *Drosophila* larval muscles involves mandatory asymmetric fusion of two types of cells, FCs and FCMs, both of which are specified from equivalence groups of myoblasts expressing different iTFs, in response to positional cues. iTF expression then becomes restricted to PCs and some FCs (Fig. 1A) [16]. iTF activation in equivalence groups raises the legitimate question of whether this influences FCM fate. To address this question, we exploited the dynamics of *col* transcription, which is activated in one PMC, maintained in one FC and re-activated in FCM nuclei incorporated into the growing DA3 myotube [35]. Our data showing that FCMs recruited in a given myofibre can originate from different equivalence groups and are equally competent to be converted to a specific FC identity, indicate that fused FCMs behave as naive myoblasts. This naive character of FCMs finds parallels with the uncommitted character of mammalian satellite cells. Mouse satellite cells specified in different anatomical locations have been shown to contribute to new fibres in a heterotopic location, though retaining distinct molecular signatures reflecting, in part, their developmental history [40]. Similarly, grafting of human myogenic progenitors into the dystrophic pharyngeal muscles of oculopharyngeal muscular dystrophy patients shows that ectopic myoblasts isolated from clinically unaffected limbic muscles are able to restore contractility to defective pharyngeal muscles [41]. Thus, *Drosophila* FCMs and mammalian satellite cells are able to contribute to different muscle fibres, irrespective of their early molecular signature. In both cases, the syncytial cell is able to reprogramme fusing cells and retain its identity.

### Transcriptional reprogramming of naive myoblast nuclei, post fusion

Previous analyses, performed at the protein expression level, led to the conclusion that muscle lineage-specific iTF expression, which determines muscle identity, reflects cross-regulations between iTFs at the PC and FC stages [12, 42]. Here, the patterns of nascent *col*, *S59* and *Kr* transcripts show that propagation of transcriptional identity from the FC to recruited FCM nuclei depends upon the iTF and/or muscle lineage, and that there is not one pattern to explain this process. In the case of *col* in the DA3 muscle or *tailup/islet1* in dorsal muscles, activation of transcription relies upon one CRM, while propagation to syncytial nuclei involves another, ‘late’ CRM mediating direct autoregulation by the imported iTF [31, 43]. In the case of *Kr*, a single muscle CRM has been identified. This fits with the restriction of *Kr* transcription to the FC nucleus and arrest earlier than observed for iTF genes displaying auto-regulatory CRMs. Future characterisation of multiple iTF CRMs differentially active in different muscle lineages should provide a general picture of how PC/FC identity is maintained and/or propagated during muscle development, leading to a robust, stereotyped muscle pattern.

**Fig. 9:**
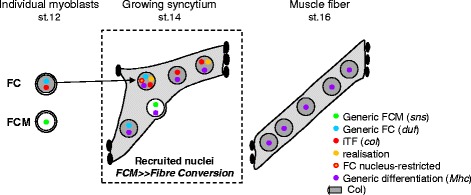
Summary model for transcriptional (re)programming of individual syncytial nuclei in a developing muscle. Before fusion, stage 12, all founder cells (FCs) transcribe generic FC genes (*duf*, *blue dot*), and fusion competent myoblasts (FCMs) transcribe FCM-specific genes (*sns*, *green dot*). Each FC expresses specific identity transcription factors (iTFs; e.g. Col, *grey*) and transcribes iTF genes (e.g. *col*, *red dot*). In a growing muscle syncytium, stage 14, FCM gene transcription is transitorily maintained in newly fused FCM nuclei, before complete switch off and progressive activation of FC-generic transcription. Nuclear uptake of iTFs by fused FCMs is followed by iTF activation. Transcription of identity-realisation genes (e.g. *Pax*, *mspo*, *kon*, *orange dot*) is linked to iTF activation, while transcription of general muscle differentiation genes (*Mhc*), is activated and maintained in all myoblast nuclei, independently of iTF expression. Transcription of a subset of genes is restricted to the FC nucleus (e.g. *Con*, *orange dot circled* by a *red line*)

Fused FCM nuclei stop transcribing FCM-specific genes, such as *sns*, before activating generic FC genes such as *duf* (Fig. 9). One mechanism involved in this transcriptional switch is the active degradation of Lmd, present in the fusing FCMs, by the ubiqutitin ligase Mib2 [44]. Whether *duf* activation also requires degradation of TTK69, a repressor of FC fate [14], and/or import of generic FC activator TF(s), similar to import of specific iTFs, remain open questions. In addition, a potential epigenetic control of nuclei reprogramming in multinucleated cells has been scarcely addressed thus far. Mutants for *Drosophila* Sin3A, a chromatin regulator conserved from yeast to humans that serves as a scaffold protein for the Sin3/histone deacetylase complex, show muscle identity phenotypes [45], suggesting that Sin3A could sensitise certain FCs and muscles to iTF activity. Patterns of epigenetic histone markers were found to be identical in all nuclei of mouse multinucleated osteoclasts, whereas some osteoclast-specific genes were only transcribed in a subset of nuclei [46]. Further investigation on different types of multinucleated cells is certainly needed for mechanical understanding of the reprogramming of syncytial nuclei, post fusion.

### Transcriptional regulation of muscle-generic and identity genes: two independent programmes

Gene expression studies, using either mammalian C2C12 cells in culture or in vivo models of muscle regeneration, have shown that abrupt, orderly transcriptional changes occur during myogenesis [47]. However, global transcriptome analysis of cell pools produces a population average that does not distinguish between transcriptional and post-transcriptional regulatory levels. Analyses of muscle cells isolated from *Drosophila* embryos at different time points showed that recruitment of poised RNA polymerase II, which makes genes permissive for future transcription, occurs at many genes de novo, in a stage-specific way [48]. In parallel, systematic genomic-wide analyses of transcription factor binding profiles and chromatin marks has identified temporal signatures of enhancer activity in the *Drosophila* mesoderm [49, 50]. While revealing the importance of timing of enhancer/promoter activity during mesoderm development, these studies did not address the question of the dynamics of gene transcription in individual syncytial nuclei. Our analysis of nascent transcripts in individual nuclei in defined muscles has freed us from post-transcriptional regulation levels that contribute to differential mRNA accumulation, as documented for the regulation of *Mhc* by Hoi Polloi [51]. Moreover, our approach allowed the comparison of the transcriptional time interval of a general muscle differentiation gene, *Mhc*, with iTFs and identity-realisation genes in individual syncytial nuclei. In this manner, we observed a mixture of ON/OFF nuclei for iTFs and realisation genes, whose relative proportions varied between muscle lineages, suggesting a stochastic mode of activation. Inversely, we found that *Mhc* transcription is synchronously activated in all myoblast nuclei independent of muscle identity, correlating with the previous finding that Mhc is expressed in all myoblasts, independent of fusion [39]. We conclude that the generic muscle differentiation and morphological identity programmes are regulated independently of each other. Transcriptional control of the generic aspects of skeletal muscle development in vertebrates involves several transcription factors that act as muscle iTFs in *Drosophila*, including Nautilus/MyoD, Eya, Six and Col/EBF proteins [12, 52–54]. Our data in *Drosophila* plead for investigating whether muscle identity and generic differentiation programmes also run in parallel in vertebrates.

### Transcription of identity realisation genes is linked to iTF transcription

How realisation of muscle identity is integrated with generic muscle differentiation is a major question in muscle biology. Here, we compared the transcriptional dynamics of candidate morphological realisation genes in syncytial nuclei between different muscles [11]. Our data show that the differential expression of genes encoding surface protein, such as *Con*, *kon* and *mspo*, between the DA3 and DT1 muscles is controlled at the transcriptional level, via both regulation of the time interval of active transcription and the transcription initiation rate. Moreover, transcription of these genes in the DA3 muscle is restricted to nuclei transcribing *col*, i.e. reprogrammed to DA3 identity by imported Col protein (Fig. 9). However, not every *col* ^+^ nucleus transcribes realisation genes, suggesting either one of three not mutually exclusive possibilities, namely (1) higher threshold levels of imported Col protein are required for activation of realisation genes than for *col* autoregulation, thus introducing a temporal shift; (2) the burst frequency of realisation gene transcription is significantly lower than that of *col*; (3) other transcription factors, themselves regulated by Col, are involved in activation of realisation genes, as documented in the case of Col and Apterous-expressing neurons [55–57]. Finally, whether local *col* mRNA translation around *col*-transcribing nuclei could amplify variations of gene transcription among syncytial nuclei is an interesting question. We also found that transcription of realisation genes is generally independent of nucleus position within the fibre. One exception is the *Con* gene, which is transcribed in a single nucleus at a central position that we surmise is the FC nucleus. The restriction of *Con* transcription to the FC in DA3 contrasts with its propagation to other syncytial nuclei in DT1. Thus, syncytial activation is an active process regulated in a fibre-specific manner. Together with the restricted pattern of *Kr* transcription, DA3 *Con* transcription also suggests that FC nuclei can keep a transcription programme distinct from the other syncytial muscles throughout muscle development. Whether this could provide molecular cues for positioning of the neuromuscular synapse remains an open question.

The correlation in the severity of DA3 morphology defects with *col* mutations of increasing strength, or mutations in other iTFs which affect *col* transcription [12, 30], suggests that proper activation of realisation genes is dependent upon the appropriate levels of iTF activity. Our present finding that transcription of realisation genes is linked to iTF transcription strongly supports this conclusion. *Con*, *kon* and *mspo* were identified as putative direct Col targets by genome-wide ChIP-SEQ experiments [56]. This, and other genome wide data [58, 59], suggest that direct binding of different iTF combinations is responsible for the differential transcription of identity realisation genes in different muscles. Establishing whether specific CRMs integrate multiple iTF inputs, or multiple CRMs respond to individual iTFs, will be the next step towards deciphering the regulatory logic that confers each muscle its specific morphology.

## Conclusions

In conclusion, our data show that post-fusion reprogramming of naive myoblast nuclei is progressive, such that nuclei within one syncytial fibre are heterogeneous with regards to gene-specific transcription at a given time. While reprogramming seems independent of nuclear position, preliminary evidence indicates that FC nuclei can remain transcriptionally different from other nuclei throughout muscle development (Fig. 9). This novel view of the dynamics of transcriptional (re)programming of post-mitotic nuclei within syncytial cells provides a new framework for the investigation of transcriptional control of lineage diversity of multinucleated cells.

## Methods

### *Drosophila* strains

All *Drosophila melanogaster* stocks were grown and genetic crosses performed on standard medium at 25 °C. The strains used were *w* ^*118*^ as wild-type (wt), *col* ^*ECRM*^ *-H2bRFP*, *col* ^*LCRM*^ *-*moeGFP (formerly *4_0.9col-moeGFP* [60]), *S59-mcd8GFP* [23], *Kr* ^*GMR80H11*^ *-Gal4* [32], *UAS-mcd8GFP*, *UAS-LacZ*, *P{PZ}sr* ^*03999*^ (Bloomington Stock Centre). The *col* ^*1*^ strain was balanced using *CyO*,{*wg* ^*en11*^ *-lacZ*} and homozygous mutant embryos identified by absence of *lacZ* expression.

### Constructions of the *col*^*ECRM*^*-*H2B-RFP reporter line

The AttB-*col* ^*ECRM*^ *-*H2bRFP transgenic construct was obtained by inserting the *col* ^*ECRM*^ genomic fragment (formerly CRM276 [31]) into the AttB-H2bRFP plasmid. AttB-H2bRFP was made by introducing H2bRFP sequences (taken from the P13-pc5-H2b-RFP plasmid, a gift from Dr John Wallingford, UT Austin, USA) into the AttP-pS3AG transgenesis vector (Addgene #31171, Thomas Williams Lab). AttB-*col* ^*ECRM*^H2bRFP was inserted at position 49D on the second chromosome by using the ZH8 AttP integration site [61].

### In situ hybridisation and antibody staining

Embryo antibody staining and in situ hybridisation with exonic or intronic probes were as previously described [35]. The following primary antibodies were used: rabbit anti-Mef2 (1:800; from E. Furlong, EMBL, Germany), anti-β3-tubulin (1:5000; from R. Renkawitz-Pohl, Philipps Univ., Germany), anti-Lmd (1:1000; from E. Furlong), anti-GFP (1:500; Torrey Pines Biolabs), anti-RFP (1:1200; Rockland Immunochemicals), anti-LacZ CAPPEL (1:500; MP Biomedicals), anti-S59 (1:400; from M. Frasch, Erlangen, Germany), anti-Kr (1:500; from R. Pflanz, Goettingen, Germany), chicken anti-GFP (1:500; Abcam), mouse anti-Col (1:50), anti-FascII^ID4^ (1:20; Hybridoma bank), and rat anti-RFP (1:500; Chromotek). Secondary antibodies were Alexa Fluor-488, -647 and -555 conjugated antibodies (1:300; Molecular Probes). DIG- or biotin-labelled RNA probes were transcribed in vitro using T7 or SP6 RNA polymerase, from PCR-amplified DNA sequences, either cloned by in pGemTeasy or directly. The sequence of primers used is given in Additional file 19: Supplementary Materials and Methods. Optimal confocal sections were acquired on Leica SP5, Leica SPE or Zeiss 710 microscopes at 40× magnification. Projections and 3D reconstructions were made using ImageJ and Volocity (PerkinElmer) software, respectively.

### Quantification of the number of RFP^+^ nuclei

*col* ^*ECRM*^ *H2bRFP; col* ^*LCRM*^ *moeGFP*, *col* ^*ECRM*^ *H2bRFP; S59-mcd8GFP* and *col* ^*ECRM*^ *H2bRFP; Kr* ^*GMR80H11*^ *-Gal4; UAS-mcd8GFP* embryos were stained for GFP, RFP and Mef2. Optimal Z sections were acquired at × 40 objective from at least 10 different stage 15 embryos. Since Mef2 or RFP^+^ cells can be localised deeper or more superficial than particular muscles, the number of RFP^+^ and Mef2 nuclei contained in GFP stained muscles (DA1, DT1, VA2, LO1, VA2 or LT1–LT4) was determined by manual counting of Z sections, rather than Z projections or 3D reconstructions (*n* = 30 muscles). The same procedure was used to count the number of RFP^+^ nuclei in DA3, DT1 and DA2 muscles in *col* ^*ECRM*^ *H2bRFP* and *col* ^*1*^, *col* ^*ECRM*^ *H2bRFP* embryos (n > 15), stained for RFP and β3-Tub (*n* = 50 muscles). Data plots and statistical analyses were performed with Prism 5.0. using unpaired *t* test, the Mean number of nuclei ± SD was shown.

### Quantification of relative mRNA levels in DA3 and DT1 muscles

For all exonic probes, the same laser parameters were used for image acquisition, from at least six stage 15 embryos stained for β3-Tub (488), Col (647) and ISH (555). Optimal Z sections were acquired at × 40 objective and data analysed with ImageJ. For each Z stack, a Sum slices projection was generated. The region of interest (ROI) corresponding to the DA3 and DT1 muscles was manually drawn based on the muscle shape visualised by β3-Tub staining. ROIs were used on the red channel of the Sum projection to determine a mean value corresponding to the mean intensity in each ROI. Mean values acquired for the DA3 and DT1 on the same images were used as a measure of relative expression levels in these two muscles (*n* = 30 muscles).

### Quantification of the number and intensity of transcriptional dots per muscle

To determine the dynamics of iTF expression in growing syncytium (Fig. 3), *col* ^*LCRM*^ *moeGFP*, *S59-mcd8GFP* or *Kr* ^*GMR80H11*^ *-Gal4; UAS-mcd8GFP* embryos were stained for GFP (488), Col, S59 or Kr (647), respectively, and ISH (555). To quantify realisation gene expression in DA3 and DT1 (Fig. 6), *col* ^*LCRM*^ *moeGFP; S59-mcd8GFP* embryos were stained for GFP (488), Topro (647) and ISH (555). For all experiments, for each intronic probe, the same laser parameters were used for image acquisition, from at least five embryos each at stages 12, 13, 14, 15 and 16. Optimal Z stacks were acquired at × 40 objective. ImageJ was used to analyse the data. For each stack, a Sum slices projection was generated, and for each probe, a threshold was applied to the red channel to remove background and to generate a black and white image, called ‘mask of dots’ corresponding to transcription dots only. In parallel, ROIs corresponding to muscle of interest were manually drawn, based on the green channel to generate a ‘muscle mask’. The position of hybridisation dots localised in each muscle of interest was automatically determined via Image Calculator process using the AND subtraction between the mask of dots and the muscle masks. The ROI for each dot was determined via the Analyse Particles option. ROI dots in a muscle of interest were verified by comparison with dot localisation in the Z section, to manually remove ROIs corresponding to dots localised above or below the considered muscle. The dot ROIs were used on the red channel of the Sum projection to measure the intensity (IntDen, Integrated Density) of each dot and count the number of dots per muscle. Data plots and statistical analyses were performed with Prism 5.0 using unpaired *t* test. For each condition at each embryonic stage from 12 to 16, *n* = 20 muscles, except for *Kr* FISH staining (*n* = 12). Statistical analyses account for variations in dot intensities that reflect the discontinuous character of transcription [29]. The same images were used to manually count the number of nuclei. For Fig. 3, the number of nuclei in the DA3 were counted using Col staining; in the DT1, LO1, VA2 and VT1, the number of nuclei were counted using S59 staining; and in the Kr-positive muscles, the number of nuclei were determined using the Kr staining. In Fig. 6, the number of nuclei in DA3 and DT1 muscles at stages 12, 13, 14, 15 and 16 were counted based on GFP and Topro staining. The Total Integrated Density per fibre (Fig. 6) corresponds to the sum of the Integrated Density of dots in one muscle. The mean value ± SEM of the Total Integrated Density in DA3 and DT1 at various stages were calculated.

### Quantification of the number of transcriptional dots in double FISH experiments

To quantify gene expressions in DA3, wt embryos were stained for double ISH (555 and 488), Col (647) and DAPI. For all intronic probes, the same laser parameters were used for image acquisition, from at least four embryos each at stages 12 to 16. Optimal Z stacks were acquired at × 40 objective. The number of nuclei and the number of transcription dots were manually counted. Because intensity of transcriptional dots were not analysed in this case, no normalisation threshold was applied, and all dots were counted, even if small and/or weak. The DA3 muscle was identified with the Col staining; the number of nuclei in the DA3 was counted with the DAPI staining. DAPI staining was also used to identify nuclei with only one or two transcription dots. Data plots were performed with Prism 5.0 for each embryonic stage (*n* = 12 muscles).

### Quantification of the relative distributions of nuclei and transcriptional dots in DA3 muscles

For each intronic probe, at least 10 *col* ^*LCRM*^ *moeGFP* late stage 14 embryos stained for GFP (488), Col (647) and ISH (555) were recorded. DA3 muscles (*n* = 25) were oriented along the AP axis and cropped to generate a minimal DA3 bounding rectangle. For each cropped Z stack, a max intensity slices projection was generated and the ROI corresponding to the DA3 was manually drawn based on the green channel. ROIs corresponding to DA3 nuclei were drawn based on Col and moeGFP staining. A threshold was applied to the red channel (ISH staining) to generate a mask of dots, used to automatically determine ROI dots via *Analyse Particles*. ROI dots and ROI nuclei localised outside the DA3 were manually removed to only keep DA3 dots ROI or DA3 nuclei ROI. To determine the spatial coordinates of DA3, the dots and the nuclei, the following values were used: for the DA3, the DA3 Area, Centroid position (X^DA3^; Y^DA3^) and coordinates of the Bounding Rectangle (BR: X^BR^; Y^BR^; Width; Height); for the dots and nuclei, the position of their Centroid (X; Y) (Additional file 17: Figure S5). Excel was used for all of the following calculations. The coordinates of DA3 BRant (X^BRant^ = X^BR^; Y^BRant^ = Y^BR^ + Height) and BRpost (X^BRpost^ = X^BR^ + Width; Y ^BRpost^ = Y^BR^) were calculated. For each BRant, BRpost and dot centroid position, their distance to the corresponding DA3 centroid was calculated to centre the coordinates on this point (ΔX = X – X^DA3^; ΔY = – (Y – Y^DA3^)). All the values were reported to a fixed DA3 area to standardise the data (standΔX = ΔX × 100/area; standΔY = ΔY × 100/area). The graphs in Fig. 8 show standardised positions of each dot and the mean position of BRant and BRpost, centred on the DA3 centroid (n^DA3^ = 25). A DA3 shape was added on each graph to better visualise the dot distribution.

## Additional files

Additional file 1: Table S1. Number of Mef2- and RFP-positive nuclei in dorsolateral, lateral and ventral muscles in stage 15 embryos. The numbers of RFP- and Mef2-positive nuclei were determined in the DA3 using *col* ^*ECRM*^ *-H2bRFP; col* ^*LCRM*^ *-moeGFP* embryos; in the DT1, the LO1 and the VA2, using *col* ^*ECRM*^ *-H2bRFP; S59-mcd8GFP* embryos; and in the four LT muscles, using *col* ^*ECRM*^ *-H2bRFP; UAS-mcd8GFP ; Kr* ^*GMR80H11*^ *-Gal4* embryos, stained for GFP, RFP and Mef2. For each muscle, the average number of nuclei ± standard deviation, minimum and maximum number of nuclei are given (*n* = 30). (PDF 287 kb)

Additional file 2: Figure S1. Identification of a *Kr* CRM active in the LTs and VA2 muscles. (A) Chromosome (2R: 25,200,000–25,237,000) showing the *Kr* genomic region with the transcribed region represented by a blue box and all GMR tested by grey lines; adapted from Flybase GBrowse (http://flybase.org/). The overlapping GMR80H07 and GMR80H11 driving reporter expression in LTs and VA2 muscles are indicted by orange lines. (B, C) Stage 16 *Kr* ^*GMR80H07*^ *-Gal4; UAS-LacZ* (B) and *Kr* ^*GMR80H11*^ *-Gal4; UAS-LacZ* (C) embryos stained for LacZ, illustrating LacZ expression in somatic muscles. (PDF 862 kb)

Additional file 3: Table S2. Number of RFP positive nuclei in the DA1, DT1 and DA2 muscles in stage 15 wt and *col* ^*1*^ mutant embryos. The RFP-positive nuclei in the DA3, DA2 and DT1 muscles were counted in *col* ^*ECRM*^ *-H2bRFP* and *col* ^*1*^, *col* ^*ECRM*^ *-H2bRFP* embryos, stained for RFP and β3-Tub to visualise muscle shape. In *col* mutant embryos, the DA3 orients like a DA2 muscle (DA3 > DA2). For each muscle, the average number of nuclei ± standard deviation, the minimum and maximum number of nuclei are given (*n* = 50). (PDF 9 kb)

Additional file 4: Table S3. Dynamics of *col* and *S59* transcription during muscle differentiation. In the DA3, the number of nuclei and the number of *col* transcription dots were counted in *col* ^*LCRM*^ *-moeGFP* embryos, using FISH with *col* intronic probes, coupled with GFP and Col staining. In the DT1, LO1, VA2 and VT1, the number of nuclei and the number of *S59* transcription dots were counted in *S59-mcd8GFP* embryos, using FISH with *S59* intronic probes, coupled with GFP and S59 staining. For each muscle and stage, the mean number of dots (or nuclei) ± standard deviation, and minimum and maximum numbers of dots (or nuclei) are given (*n* = 20). The same samples were also used for Additional file 6: Table S4. (PDF 173 kb)

Additional file 5: Figure S2. Dynamics of *col*, *S59* and *Kr* transcription during muscle differentiation. (A) Stage 14 *col* ^*LCRM*^ *-moeGFP* embryo stained for GFP (green) and Col (blue), coupled with FISH of nascent *col* transcripts (red), four adjacent segments are shown. (B) Intensity of each *col* transcriptional dots in DA3, at stages 12, 13, 14, 15 and 16. Each dot is represented by one open circle; the bar graphs show the mean values and SDs. (C) Box plots showing the number of *S59* transcription dots (orange) and nuclei (grey) in the DT1, LO1, VA2 and VT1 muscles, at stages 12, 13, 14, 15 and 16. (D) Percentage of nuclei transcribing *S59* in the DT1, VA2 and VT1 muscles, at stage 15. Bar graphs show the mean percentage of active nuclei and error bars correspond to the SEM; statistical analyses were performed using Pearson’s χ^2^ test. Asterisks show the significance of variation (ns: not significant; (***): *P* value < 0.001). (E) Intensity of each *S59* transcriptional dot in DT1, LO1, VA2 and VT1 muscles, at stages 12, 13, 14, 15 and 16. (F) Box plots showing the numbers of *Kr* transcription dots (yellow) and nuclei (grey) in the DA1, DO1, LL1, LT2, LT4 and VA2 muscles, at stages 12, 13, early 14, late 14 and 15. (G) Intensity of each *Kr* transcriptional dot in DA1, DO1, LL1, LT2, LT4 and VA2, at stages 12, 13, early 14 and late 14. (PDF 2742 kb)

Additional file 6: Table S4. Integrated density of *col* and *S59* transcriptional dots during muscle differentiation. For each muscle and stage, the mean intensity of *col* transcriptional dots in the DA3 and *S59* transcriptional dots in the DT1, LO1, VA2 and VT1 muscles ± standard deviation, and the minimum and maximum intensity are given (*n* = 20). Same embryo samples as in Additional file 4: Table S3. (PDF 21 kb)

Additional file 7: Table S5. Dynamics of *Kr* transcription during muscle differentiation. The number of nuclei and the number of *Kr* transcription dots were counted in *Kr* ^*GMR80H11*^ *-Gal4; UAS-mcd8GFP* embryos, using FISH with *Kr* intronic probe coupled with GFP and Kr staining. For each muscle and stage, the mean number of dots (or nuclei) ± standard deviation, and minimum and maximum numbers of dots (or nuclei) are given. The number of nuclei is determined using the Kr staining, whose detection decreases from early stage 14 until the end (*n* = 12, except for conditions indicated with an asterisk, corresponding to stages where determination of nuclei number is limited due to the loss of Kr staining). The same samples were also used for Additional file 8: Table S6. (PDF 179 kb)

Additional file 8: Table S6. Integrated density of *Kr* transcriptional dots during muscle differentiation. For each muscle and stage, the mean intensity of *Kr* transcriptional dots in the DA1, DO1, LL1, LT2, LT4 and VA2 muscles ± standard deviation, and the minimum and maximum intensity are given. Same embryo samples as in Additional file 7: Table S5. (PDF 21 kb)

Additional file 9: Figure S3. Separate transcription of *col* and *duf* in DA3 syncytial nuclei. (A–C) FISH against nascent *duf* transcripts (green) coupled to Mef2 (red) and Col (blue), immunostaining of all muscle and DA3 nuclei, respectively, in stage 13, 14 and 15 wt embryos. (D) Double FISH of nascent *col* (red) and *duf* (green) transcripts in stage 14 wt embryos immunostained for Col (blue); (D’) Col staining alone; (D”) same as (D), showing DAPI staining (blue) of all nuclei. Single Z sections are shown. The yellow arrow points to a nucleus with low Col protein level transcribing *duf* and not *col*. (E) Box plots showing the number of nuclei transcribing either *col*, *duf* or both, or either *col* or *sns*, relative to the total number of DA3 nuclei. (PDF 2537 kb)

Additional file 10: Table S7. FCM to FC/fibre programme conversion: dynamics of *col* ^*+*^ *duf* and *col* ^*+*^ *sns* transcription in a growing DA3 muscle. The number of nuclei and the number of *col* and *duf* or *col* and *sns* transcription dots were counted in wt embryos, using double FISH with *col* and *duf* or *col* and *sns* intronic probes coupled with Col and DAPI. The DA3 muscle was identified with the Col staining; the number of nuclei in the DA3 was counted with the DAPI staining. DAPI staining was also used to identify nuclei with only one or two transcription dots. For each muscle and stage, the mean number of dots (or nuclei) ± standard deviation, and minimum and maximum numbers of dots (or nuclei) are given (*n* = 12). (PDF 163 kb)

Additional file 11: Figure S4. Expression of generic differentiation and identity realisation genes in DA3 and DT1. (A) Stage 15 *col* ^*LCRM*^ *-moeGFP* embryos stained for GFP (green) and β3-tub (blue) coupled to FISH against *Mhc* mRNA (red). (A’) red channel only. (B) Numbers of nuclei in the DA3 and DT1 muscles, counted in *col* ^*LCRM*^ *-moeGFP; S59-mcd8GFP* embryos stained for GFP and Topro, at stages 12, 13, 14, 15 and 16. For each condition, the mean number of nuclei ± standard deviation is shown (n = 20). The same embryo samples were used for Fig. 6. (C, D, F, H, J) Stage 15 wt embryos stained for β3-tub (green) and Col (blue), coupled to FISH of *duf* (C), *Pax* (D), *mspo* (F), *kon* (H) and *Con* (J) mRNA (red); (C’, D’, F’, H’, J’) red channel only. A schematic representation of mRNA expression in DA3 and DT1 is shown on the right, with grey intensity reflecting the level of mRNA accumulation. (E, G, I, K) Stage 14 wt embryos stained for Mef2 (red) and Col (blue) coupled to FISH against nascent *Pax* (E), *mspo* (G), *kon* (I) and *Con* (K) transcripts (green). The DA3 position is identified by Col staining (blue) and DT1 position is surrounded by a grey dotted line. (PDF 4341 kb)

Additional file 12: Table S8. Dynamics of *col* and generic (*Mhc*) or realisation gene (*kon*, *mspo*) transcription in a growing DA3 muscle. The number of nuclei and the number of *col* and *Mhc*, *kon* or *mspo* transcription dots were counted in wt embryos, using double FISH with *col* and *Mhc*, *kon* or *mspo* intronic probes coupled with Col and DAPI. The DA3 muscle was identified with the Col staining; the number of nuclei in the DA3 was counted with the DAPI staining. DAPI staining was also used to identify nuclei with only one or two transcription dots. For each muscle and stage, the mean number of dots (or nuclei) ± standard deviation, and minimum and maximum numbers of dots (or nuclei) are given (*n* = 12). (PDF 173 kb)

Additional file 13: Table S9. Relative mRNA levels of *duf* and realisation genes. Relative levels of *duf*, *Pax*, *mspo*, *kon* and *Con* mRNA in DA3 and DT1 muscles were measured in wt embryos using FISH with exonic probes, and β3-Tub to visualise the muscle shape. The FISH mean intensity ± standard deviation is given for each muscle (*n* = 30). (PDF 7 kb)

Additional file 14: Table S10. Numbers of nuclei and number of transcriptional dots of *duf* and realisation genes in DA3 and DT1 muscles at embryonic stages 12 to 16. The numbers of nuclei and transcriptional dots in the DA3 and DT1 muscles were counted in *col* ^*LCRM*^ *-moeGFP; S59-mcd8GFP* embryos using FISH with intronic probes, coupled with GFP and Topro staining. For each muscle and stage, the mean number of dots (or nuclei) ± standard deviation, and minimum and maximum numbers of dots (or nuclei) are given (*n* = 20). The same samples were also used for Additional files 15 and 16: Tables S11 and S12. (PDF 174 kb)

Additional file 15: Table S11. Integrated density of transcriptional dots of *duf* and realisation genes in DA3 and DT1 at stage 12 to 16. For each muscle and stage, the mean intensity of transcriptional dots in the DA3 and DT1 muscles ± standard deviation, and the minimum and maximum intensity are given for each probe (*n* = 20). Same embryo samples as in Additional files 14 and 16: Tables S10 and S12. (PDF 169 kb)

Additional file 16: Table S12. Total integrated density per fibre of *duf* and realisation genes at stages 12 to 16. For each stage, the total intensity of transcriptional dots in the DA3 and DT1 muscles and the average of total intensity ± SEM are given (*n* = 20). Same embryo samples as Additional files 14 and 15: Tables S10 and S11. (PDF 156 kb)

Additional file 17: Figure S5. Quantifying the repartition of transcriptional dots in DA3 subdomains: The methodology. See Methods for details. (PDF 317 kb)

Additional file 18: Table S13. Percentage of dots and nuclei localised in the antero-ventral, central and postero-dorsal DA3 subdomains. Spatial coordinates of nuclei and transcriptional dots were acquired on *col* ^*LCRM*^ *-moeGFP* late stage 14 embryos stained for GFP and Col. FISH were with intronic probes. For each gene, the percentage of dots localised in antero-ventral (Relative Delta Y < –1), postero-dorsal (Relative Delta X > 1) or central (Relative Delta X < 1 and Relative Delta Y > –1) position are shown; 25 muscles were analysed for each condition, the corresponding total number of spots (or nuclei) is indicated. (PDF 145 kb)

Additional file 19: Supplementary Material and Methods. 5’-3’ sequence of the oligonucleotide primers used for making probes for FISH. (PDF 527 kb)

## Abbreviations

col: collier
Con: connectin
CRM: cis-regulatory module
duf: dumbfounded/kin of irre
FC: founder cell
FCM: fusion competent myoblast
iTF: identity transcription factor
kon: kon*-*tiki/perdido
Kr: Krüppel
lmd: lameduck
LT: lateral transverse
Mhc: myosin heavy chain
mspo: M-spondin
nau: nautilus/MYOD
Pax: Paxillin
PC: progenitor cell
PMC: promuscular clusters
sns: sticks and stones.
