# Supplementary material for: Dynamics of transcriptional (re)-programming of syncytial nuclei in developing muscles
Source: BMC Biol. 2017 Jun 9;15:48. doi: 10.1186/s12915-017-0386-2 (PMC5466778; doi:10.1186/s12915-017-0386-2)
Supplement: Supplementary file 1 — Number of Mef2- and RFP-positive nuclei in dorsolateral, lateral and ventral muscles in stage 15 embryos. The numbers of RFP- and Mef2-positive nuclei were determined in the DA3 using col ECRM -H2bRFP; col LCRM -moeGFP embryos; in the DT1, the LO1 and the VA2, using col ECRM -H2bRFP; S59-mcd8GFP embryos; and in the four LT muscles, using col ECRM -H2bRFP; UAS-mcd8GFP ; Kr GMR80H11 -Gal4 embryos, stained for GFP, RFP and Mef2. For each muscle, the average number of nuclei ± standard deviation, minimum and maximum number of nuclei are given (n = 30). (PDF 287 kb) [file 12915_2017_386_MOESM1_ESM.pdf]

Table S1: Number of Mef2 and RFP positive nuclei in dorso-lateral, lateral and ventral muscles in stage 15 embryos.

	**DA3**		**DT1**		**LO1**		**VA2**	
	Mef2	RFP	Mef2	RFP	Mef2	RFP	Mef2	RFP
Mean ± S.D.	11,6 ± 1,04	5,57 ± 2,60	8,60 ± 0,86	3,53 ± 1,70	4,63 ± 0,67	0,77 ± 1,04	10,03 ± 1,13	0,13 ± 0,35
Minimum	10	0	6	0	3	0	8	0
Maximum	14	11	10	6	6	4	11	1

	**LT1**		**LT2**		**LT3**		**LT4**	
	Mef2	RFP	Mef2	RFP	Mef2	RFP	Mef2	RFP
Mean ± S.D.	4,93 ± 0,91	0,33 ± 0,61	4,90 ± 1,03	0,33 ± 0,61	4,97 ± 0,85	0,13 ± 0,35	4,13 ± 0,86	0,23 ± 0,50
Minimum	3	0	3	0	3	0	2	0
Maximum	7	2	7	2	7	1	6	2
