# Supplementary figures and images for: Dynamics of transcriptional (re)-programming of syncytial nuclei in developing muscles
Source: BMC Biol. 2017 Jun 9;15:48. doi: 10.1186/s12915-017-0386-2 (PMC5466778; doi:10.1186/s12915-017-0386-2)

**Figure S1 (related to Fig 1)**

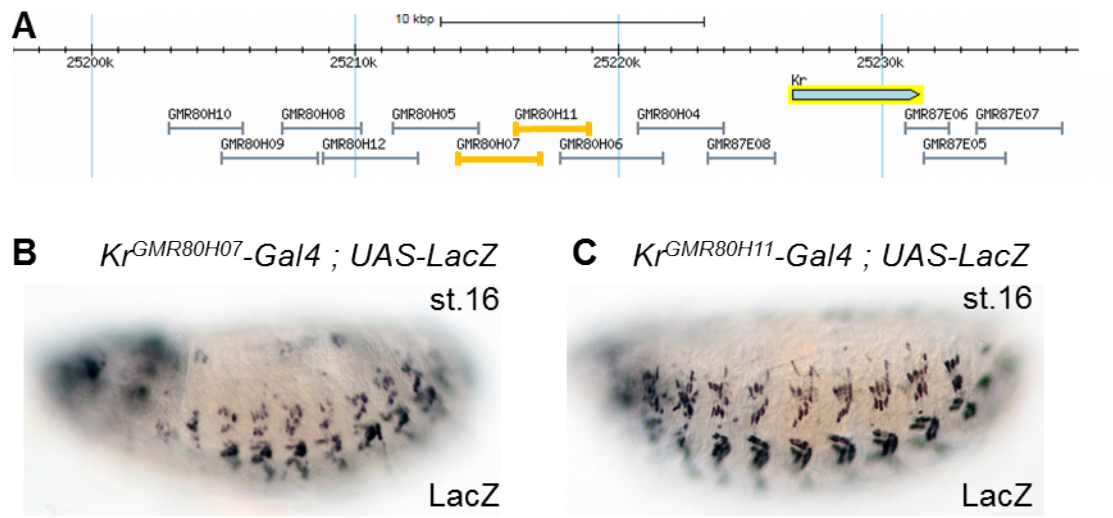

Supplement: Supplementary file 2 — Identification of a Kr CRM active in the LTs and VA2 muscles. (A) Chromosome (2R: 25,200,000–25,237,000) showing the Kr genomic region with the transcribed region represented by a blue box and all GMR tested by grey lines; adapted from Flybase GBrowse (http://flybase.org/). The overlapping GMR80H07 and GMR80H11 driving reporter expression in LTs and VA2 muscles are indicted by orange lines. (B, C) Stage 16 Kr GMR80H07 -Gal4; UAS-LacZ (B) and Kr GMR80H11 -Gal4; UAS-LacZ (C) embryos stained for LacZ, illustrating LacZ expression in somatic muscles. (PDF 862 kb) [file 12915_2017_386_MOESM2_ESM.pdf]

Figure S2 (related to Fig 3)

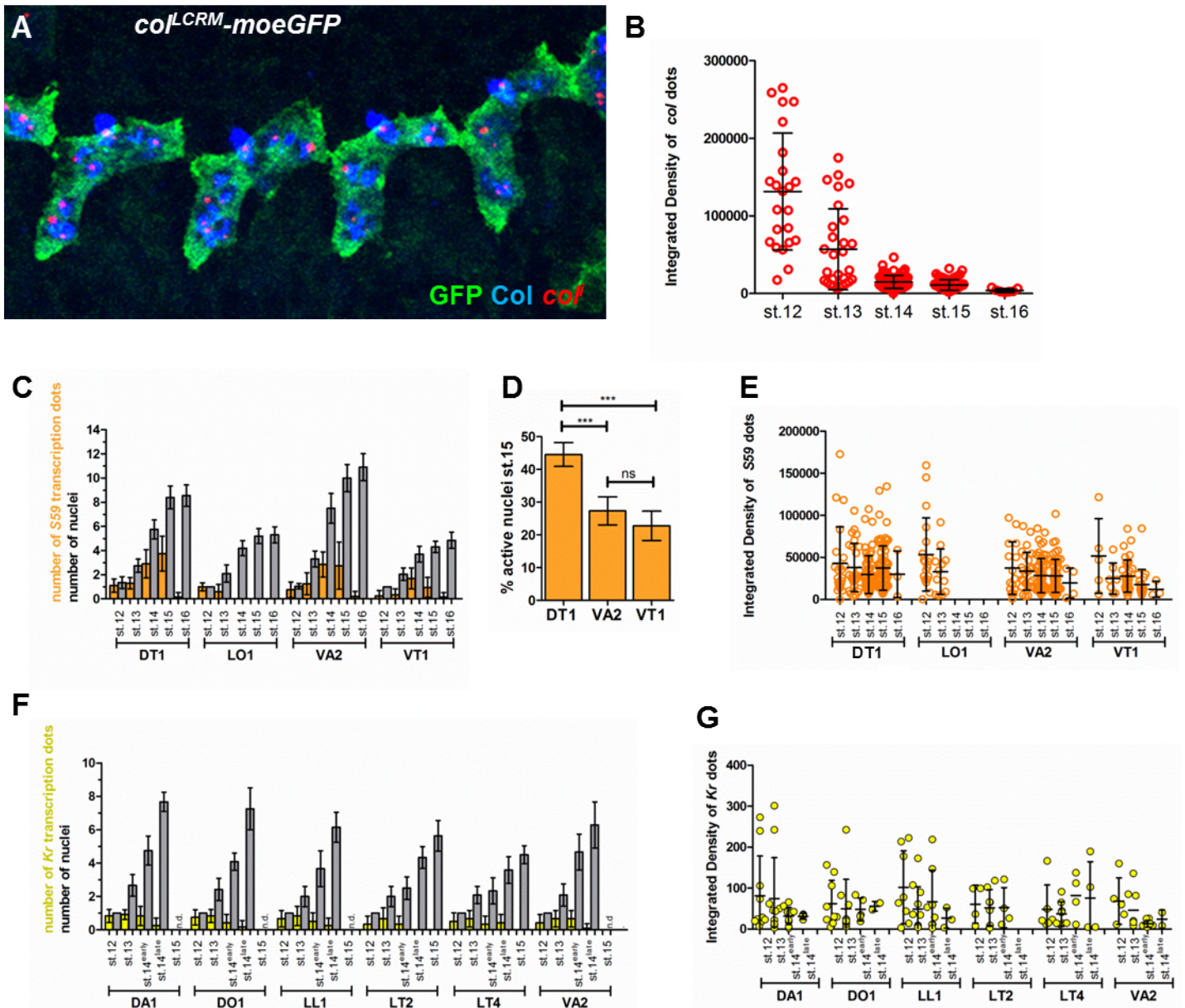

Supplement: Supplementary file 5 — Dynamics of col, S59 and Kr transcription during muscle differentiation. (A) Stage 14 col LCRM -moeGFP embryo stained for GFP (green) and Col (blue), coupled with FISH of nascent col transcripts (red), four adjacent segments are shown. (B) Intensity of each col transcriptional dots in DA3, at stages 12, 13, 14, 15 and 16. Each dot is represented by one open circle; the bar graphs show the mean values and SDs. (C) Box plots showing the number of S59 transcription dots (orange) and nuclei (grey) in the DT1, LO1, VA2 and VT1 muscles, at stages 12, 13, 14, 15 and 16. (D) Percentage of nuclei transcribing S59 in the DT1, VA2 and VT1 muscles, at stage 15. Bar graphs show the mean percentage of active nuclei and error bars correspond to the SEM; statistical analyses were performed using Pearson’s χ2 test. Asterisks show the significance of variation (ns: not significant; (***): P value < 0.001). (E) Intensity of each S59 transcriptional dot in DT1, LO1, VA2 and VT1 muscles, at stages 12, 13, 14, 15 and 16. (F) Box plots showing the numbers of Kr transcription dots (yellow) and nuclei (grey) in the DA1, DO1, LL1, LT2, LT4 and VA2 muscles, at stages 12, 13, early 14, late 14 and 15. (G) Intensity of each Kr transcriptional dot in DA1, DO1, LL1, LT2, LT4 and VA2, at stages 12, 13, early 14 and late 14. (PDF 2742 kb) [file 12915_2017_386_MOESM5_ESM.pdf]

Figure S3 (related to Fig 4)

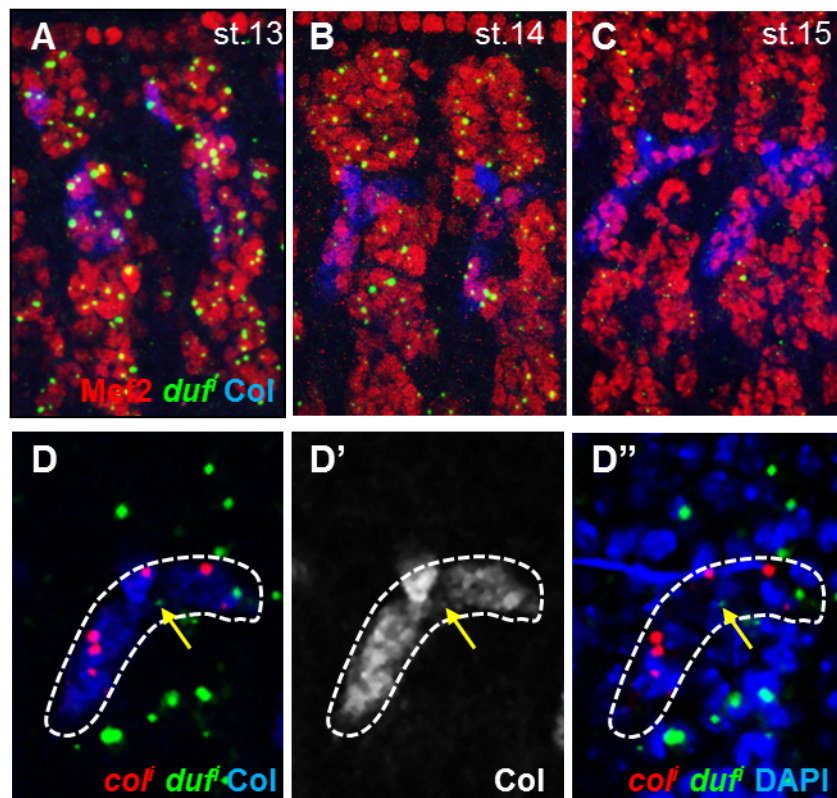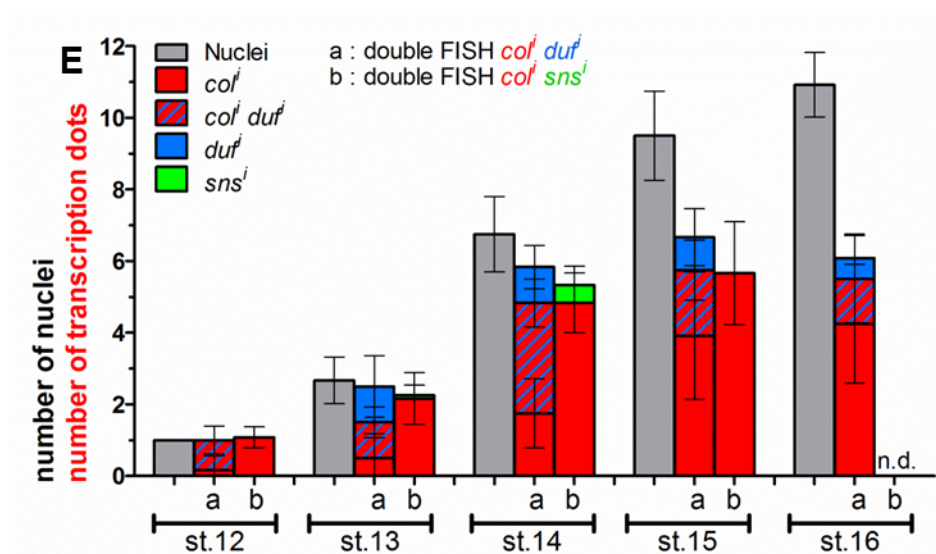

Supplement: Supplementary file 9 — Separate transcription of col and duf in DA3 syncytial nuclei. (A–C) FISH against nascent duf transcripts (green) coupled to Mef2 (red) and Col (blue), immunostaining of all muscle and DA3 nuclei, respectively, in stage 13, 14 and 15 wt embryos. (D) Double FISH of nascent col (red) and duf (green) transcripts in stage 14 wt embryos immunostained for Col (blue); (D’) Col staining alone; (D”) same as (D), showing DAPI staining (blue) of all nuclei. Single Z sections are shown. The yellow arrow points to a nucleus with low Col protein level transcribing duf and not col. (E) Box plots showing the number of nuclei transcribing either col, duf or both, or either col or sns, relative to the total number of DA3 nuclei. (PDF 2537 kb) [file 12915_2017_386_MOESM9_ESM.pdf]

Figure S4 (related to Fig 5 and Fig.6)

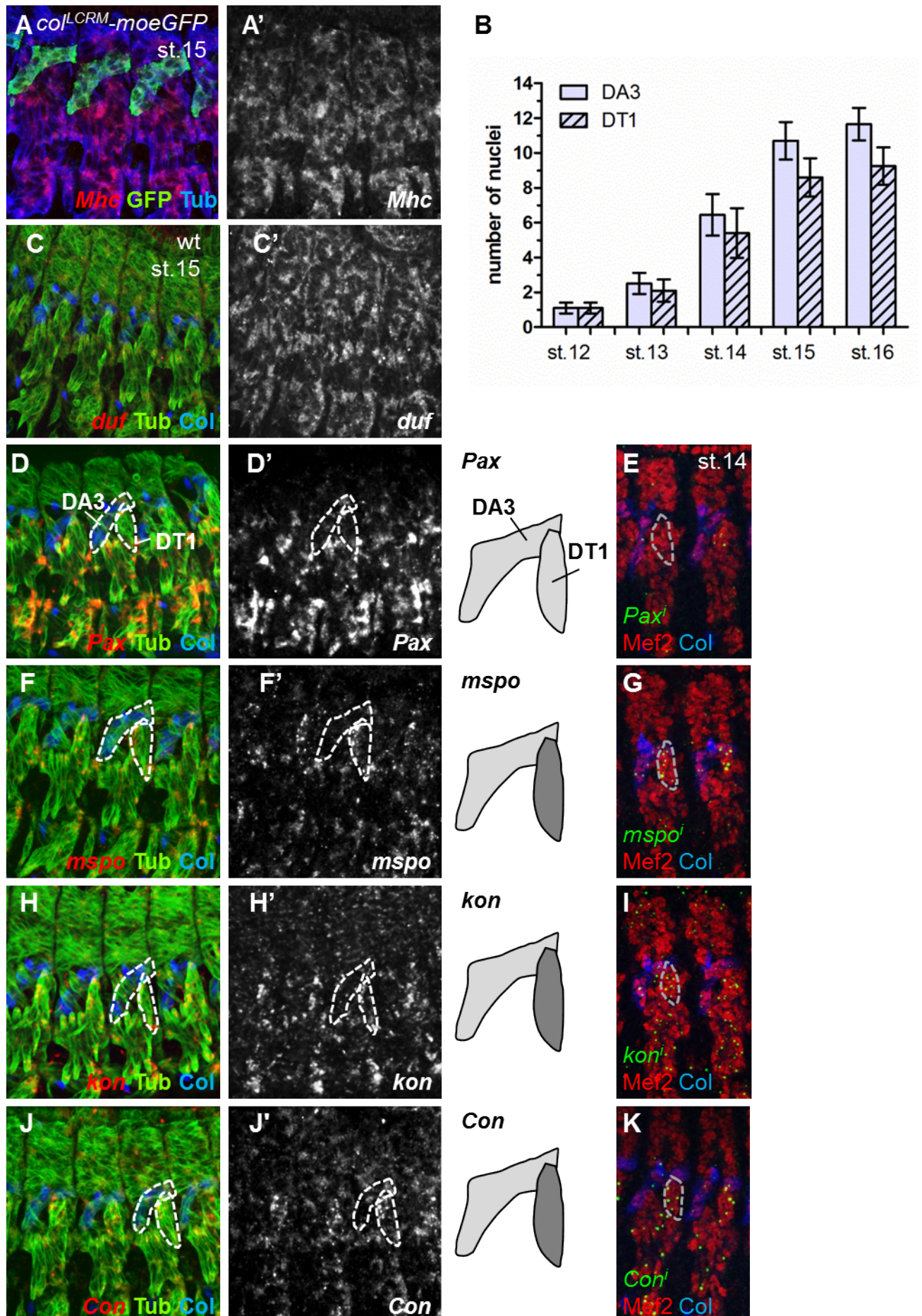

Supplement: Supplementary file 11 — Expression of generic differentiation and identity realisation genes in DA3 and DT1. (A) Stage 15 col LCRM -moeGFP embryos stained for GFP (green) and β3-tub (blue) coupled to FISH against Mhc mRNA (red). (A’) red channel only. (B) Numbers of nuclei in the DA3 and DT1 muscles, counted in col LCRM -moeGFP; S59-mcd8GFP embryos stained for GFP and Topro, at stages 12, 13, 14, 15 and 16. For each condition, the mean number of nuclei ± standard deviation is shown (n = 20). The same embryo samples were used for Fig. 6. (C, D, F, H, J) Stage 15 wt embryos stained for β3-tub (green) and Col (blue), coupled to FISH of duf (C), Pax (D), mspo (F), kon (H) and Con (J) mRNA (red); (C’, D’, F’, H’, J’) red channel only. A schematic representation of mRNA expression in DA3 and DT1 is shown on the right, with grey intensity reflecting the level of mRNA accumulation. (E, G, I, K) Stage 14 wt embryos stained for Mef2 (red) and Col (blue) coupled to FISH against nascent Pax (E), mspo (G), kon (I) and Con (K) transcripts (green). The DA3 position is identified by Col staining (blue) and DT1 position is surrounded by a grey dotted line. (PDF 4341 kb) [file 12915_2017_386_MOESM11_ESM.pdf]

Figure S5 (related to Fig 8)

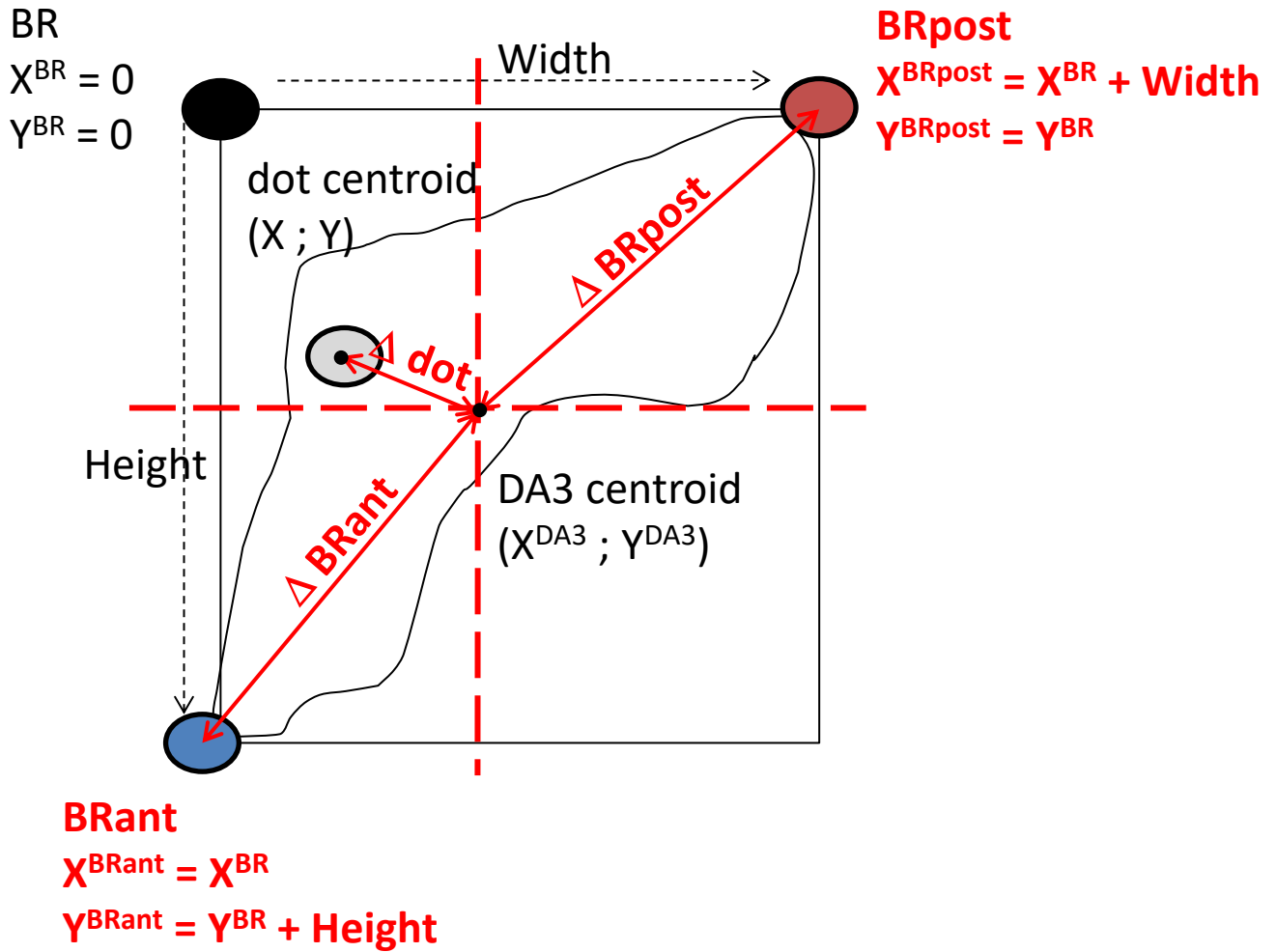

Supplement: Supplementary file 17 — Quantifying the repartition of transcriptional dots in DA3 subdomains: The methodology. See Methods for details. (PDF 317 kb) [file 12915_2017_386_MOESM17_ESM.pdf]
