# Supplementary material for: Dynamics of transcriptional (re)-programming of syncytial nuclei in developing muscles
Source: BMC Biol. 2017 Jun 9;15:48. doi: 10.1186/s12915-017-0386-2 (PMC5466778; doi:10.1186/s12915-017-0386-2)
Supplement: Supplementary file 3 — Number of RFP positive nuclei in the DA1, DT1 and DA2 muscles in stage 15 wt and col 1 mutant embryos. The RFP-positive nuclei in the DA3, DA2 and DT1 muscles were counted in col ECRM -H2bRFP and col 1, col ECRM -H2bRFP embryos, stained for RFP and β3-Tub to visualise muscle shape. In col mutant embryos, the DA3 orients like a DA2 muscle (DA3 > DA2). For each muscle, the average number of nuclei ± standard deviation, the minimum and maximum number of nuclei are given (n = 50). (PDF 9 kb) [file 12915_2017_386_MOESM3_ESM.pdf]

Table S2: Number of RFP positive nuclei in the DA1, DT1 and DA2 muscles in stage 15 wt and col^{1} mutant embryos.

	**DA3**	**DA3>DA2**	**DA2**		**DT1**	
	wt	col^{1}	wt	col^{1}	wt	col^{1}
Mean ± S.D.	4.86 ± 1.96	2.06 ± 1.36	0.56 ± 0.73	0.40 ± 0.53	3.66 ± 1.76	3.40 ± 1.75
Minimum	1	0	0	0	0	0
Maximum	9	5	2	2	8	7
