# Supplementary material for: Dynamics of transcriptional (re)-programming of syncytial nuclei in developing muscles
Source: BMC Biol. 2017 Jun 9;15:48. doi: 10.1186/s12915-017-0386-2 (PMC5466778; doi:10.1186/s12915-017-0386-2)
Supplement: Supplementary file 4 — Dynamics of col and S59 transcription during muscle differentiation. In the DA3, the number of nuclei and the number of col transcription dots were counted in col LCRM -moeGFP embryos, using FISH with col intronic probes, coupled with GFP and Col staining. In the DT1, LO1, VA2 and VT1, the number of nuclei and the number of S59 transcription dots were counted in S59-mcd8GFP embryos, using FISH with S59 intronic probes, coupled with GFP and S59 staining. For each muscle and stage, the mean number of dots (or nuclei) ± standard deviation, and minimum and maximum numbers of dots (or nuclei) are given (n = 20). The same samples were also used for Additional file 6: Table S4. (PDF 173 kb) [file 12915_2017_386_MOESM4_ESM.pdf]

Table S3: Dynamics of *col* and *S59* transcription during muscle differentiation.

			stage 12	stage 13	stage 14	stage 15	stage 16
**DA3**	number of nuclei	Mean	1,15	2,60	6,85	9,70	10,45
		Std. Dev.	0,37	0,60	1,23	0,98	0,76
		Minimum	1	2	4	8	9
		Maximum	2	4	9	11	12
	number of *col* transcription dots	Mean	1,15	1,40	4,00	3,60	0,35
		Std. Dev.	0,37	0,50	1,34	1,70	0,81
		Minimum	0	1	2	1	0
		Maximum	2	2	7	7	3
**DT1**	number of nuclei	Mean	1,35	2,75	5,75	8,40	8,55
		Std. Dev.	0,49	0,55	0,79	0,94	0,89
		Minimum	1	2	5	7	6
		Maximum	2	4	8	10	10
	number of *S59* transcription dots	Mean	1,10	1,30	2,90	3,75	0,15
		Std. Dev.	0,55	0,47	1,17	1,45	0,37
		Minimum	0	1	1	1	0
		Maximum	2	2	5	6	1
**LO1**	number of nuclei	Mean	1,00	2,10	4,20	5,20	5,30
		Std. Dev.	0,00	0,72	0,62	0,62	0,66
		Minimum	1	1	3	4	4
		Maximum	1	3	5	6	6
	number of *S59* transcription dots	Mean	1,00	0,60	0,00	0,00	0,00
		Std. Dev.	0,32	0,60	0,00	0,00	0,00
		Minimum	0	0	0	0	0
		Maximum	2	2	0	0	0
**VA2**	number of nuclei	Mean	1,05	3,30	7,50	10,00	10,90
		Std. Dev.	0,22	0,66	1,24	1,12	1,12
		Minimum	1	2	5	8	9
		Maximum	2	4	9	12	13
	number of *S59* transcription dots	Mean	0,75	1,25	2,85	2,75	0,20
		Std. Dev.	0,64	0,91	1,04	1,94	0,41
		Minimum	0	0	2	0	0
		Maximum	2	4	5	8	1
**VT1**	number of nuclei	Mean	1,00	2,05	3,70	4,30	4,85
		Std. Dev.	0,00	0,51	0,66	0,47	0,67
		Minimum	1	1	3	4	3
		Maximum	1	3	5	5	6
	number of *S59* transcription dots	Mean	0,25	0,35	1,70	0,95	0,15
		Std. Dev.	0,44	0,49	0,86	0,83	0,37
		Minimum	0	0	0	0	0
		Maximum	1	1	3	3	1
