# Supplementary material for: Dynamics of transcriptional (re)-programming of syncytial nuclei in developing muscles
Source: BMC Biol. 2017 Jun 9;15:48. doi: 10.1186/s12915-017-0386-2 (PMC5466778; doi:10.1186/s12915-017-0386-2)
Supplement: Supplementary file 6 — Integrated density of col and S59 transcriptional dots during muscle differentiation. For each muscle and stage, the mean intensity of col transcriptional dots in the DA3 and S59 transcriptional dots in the DT1, LO1, VA2 and VT1 muscles ± standard deviation, and the minimum and maximum intensity are given (n = 20). Same embryo samples as in Additional file 4: Table S3. (PDF 21 kb) [file 12915_2017_386_MOESM6_ESM.pdf]

Table S4: Integrated density of *col* and *S59* transcriptional dots during muscle differentiation.

		stage 12	stage 13	stage 14	stage 15	stage 16
**DA3**	Mean	131498	57068	14883	10868	3912
col[i]	Std. Deviation	75347	52193	8431	6870	2329
	Minimum	17385	6781	40,59	1872	1332
	Maximum	265026	174940	46560	32195	7623
**DT1**	Mean	42963	38091	29538	37321	29974
S59[i]	Std. Deviation	43524	28688	22475	26285	27649
	Minimum	147,8	3900	6508	6869	3831
	Maximum	172737	105573	107490	134434	58916
**LO1**	Mean	53451	32979	n.d.	n.d.	n.d.
S59[i]	Std. Deviation	43542	26919			
	Minimum	257,6	4702			
	Maximum	159462	91736			
**VA2**	Mean	37168	33511	28241	27971	19623
S59[i]	Std. Deviation	31212	22505	20264	19580	17820
	Minimum	892,5	4027	5873	5044	1843
	Maximum	97009	87240	84562	101816	36774
**VT1**	Mean	51731	24844	27753	17823	12116
S59[i]	Std. Deviation	44434	18591	19191	17905	9447
	Minimum	6174	7308	4823	5621	5705
	Maximum	121658	59409	84044	84388	22965
