# Supplementary material for: Dynamics of transcriptional (re)-programming of syncytial nuclei in developing muscles
Source: BMC Biol. 2017 Jun 9;15:48. doi: 10.1186/s12915-017-0386-2 (PMC5466778; doi:10.1186/s12915-017-0386-2)
Supplement: Supplementary file 7 — Dynamics of Kr transcription during muscle differentiation. The number of nuclei and the number of Kr transcription dots were counted in Kr GMR80H11 -Gal4; UAS-mcd8GFP embryos, using FISH with Kr intronic probe coupled with GFP and Kr staining. For each muscle and stage, the mean number of dots (or nuclei) ± standard deviation, and minimum and maximum numbers of dots (or nuclei) are given. The number of nuclei is determined using the Kr staining, whose detection decreases from early stage 14 until the end (n = 12, except for conditions indicated with an asterisk, corresponding to stages where determination of nuclei number is limited due to the loss of Kr staining). The same samples were also used for Additional file 8: Table S6. (PDF 179 kb) [file 12915_2017_386_MOESM7_ESM.pdf]

Table S5: Dynamics of *Kr* transcription during muscle differentiation.

			stage 12	stage 13	stage 14^{early}	stage 14^{late}	stage 15
**DA1**	number of nuclei	Mean	1,00	2,75	4,58	7,67*	n.d.
		Std. Dev.	0,00	0,62	0,79	0,58	
		Minimum	1	2	3	7	
		Maximum	1	4	6	8	
	number of *Kr* transcription dots	Mean	0,83	0,92	0,83	0,25	0,00
		Std. Dev.	0,39	0,29	0,58	0,45	0,00
		Minimum	0	0	0	0	0
		Maximum	1	1	2	1	1
**DO1**	number of nuclei	Mean	1,00	2,50	4,17	7,25*	n.d.
		Std. Dev.	0,00	0,67	0,58	1,26	
		Minimum	1	1	3	6	
		Maximum	1	3	5	9	
	number of *Kr* transcription dots	Mean	0,75	0,83	0,42	0,17	0,00
		Std. Dev.	0,45	0,39	0,51	0,39	0,00
		Minimum	0	0	0	0	0
		Maximum	1	1	1	1	0
**LL1**	number of nuclei	Mean	1,00	2,00	3,58	6,14*	n.d.
		Std. Dev.	0,00	0,60	0,79	0,90	
		Minimum	1	1	2	5	
		Maximum	1	3	5	7	
	number of *Kr* transcription dots	Mean	0,67	0,83	0,50	0,25	0,00
		Std. Dev.	0,49	0,58	0,52	0,45	0,00
		Minimum	0	0	0	0	0
		Maximum	1	2	1	1	0
**LT2**	number of nuclei	Mean	1,00	2,00	2,58	4,33	5,75*
		Std. Dev.	0,00	0,60	0,67	0,65	1,04
		Minimum	1	1	1	3	4
		Maximum	1	3	3	5	7
	number of *Kr* transcription dots	Mean	0,33	0,67	0,33	0,00	0,00
		Std. Dev.	0,49	0,65	0,49	0,00	0,00
		Minimum	0	0	0	0	0
		Maximum	1	2	1	0	0
**LT4**	number of nuclei	Mean	1,00	2,08	2,33	3,58	4,63*
		Std. Dev.	0,00	0,51	0,78	0,79	0,74
		Minimum	1	1	1	3	4
		Maximum	1	3	4	5	6
	number of *Kr* transcription dots	Mean	0,50	0,67	0,33	0,42	0,00
		Std. Dev.	0,52	0,49	0,49	0,51	0,00
		Minimum	0	0	0	0	0
		Maximum	1	1	1	1	0
**VA2**	number of nuclei	Mean	1,00	2,25	4,58	6,29*	n.d.
		Std. Dev.	0,00	0,62	0,90	1,38	
		Minimum	1	1	3	5	
		Maximum	1	3	6	8	
	number of *Kr* transcription dots	Mean	0,42	0,67	0,67	0,08	0,00
		Std. Dev.	0,51	0,49	0,49	0,29	0,00
		Minimum	0	0	0	0	0
		Maximum	1	1	1	1	0
