# Supplementary material for: Dynamics of transcriptional (re)-programming of syncytial nuclei in developing muscles
Source: BMC Biol. 2017 Jun 9;15:48. doi: 10.1186/s12915-017-0386-2 (PMC5466778; doi:10.1186/s12915-017-0386-2)
Supplement: Supplementary file 8 — Integrated density of Kr transcriptional dots during muscle differentiation. For each muscle and stage, the mean intensity of Kr transcriptional dots in the DA1, DO1, LL1, LT2, LT4 and VA2 muscles ± standard deviation, and the minimum and maximum intensity are given. Same embryo samples as in Additional file 7: Table S5. (PDF 21 kb) [file 12915_2017_386_MOESM8_ESM.pdf]

Table S6: Integrated density of *Kr* transcriptional dots during muscle differentiation.

		stage 12	stage 13	stage 14^{early}	stage 14^{late}
**DA1**	Mean	81,11	74,09	31,94	31,08
Kr^i	Std. Deviation	97,47	100,3	20,81	7,213
	Minimum	6,635	2,813	2,452	22,86
	Maximum	273	301,1	65,78	36,35
**DO1**	Mean	62,14	49,81	47,92	55,53
Kr^i	Std. Deviation	57	71,76	27,76	11,83
	Minimum	5,049	5,842	18,9	47,17
	Maximum	156,4	242,5	83,37	63,9
**LL1**	Mean	102,1	48,83	66,44	26,52
Kr^i	Std. Deviation	89,26	54,52	77,03	24,49
	Minimum	3,029	3,822	2,308	3,75
	Maximum	222,6	172,7	218,7	52,43
**LT2**	Mean	60,8	51,54	52,47	0
Kr^i	Std. Deviation	46,15	44,8	48,7	
	Minimum	7,645	2,669	11,18	
	Maximum	99,6	118,4	121,3	
**LT4**	Mean	47,96	37,06	81,86	75,44
Kr^i	Std. Deviation	59,61	29,36	55,46	88,87
	Minimum	12,33	11,4	11,25	4,688
	Maximum	166,6	91,38	137,5	189,5
**VA2**	Mean	68,24	46,03	12,87	24,38
Kr^i	Std. Deviation	56,66	48,36	8,173	22,54
	Minimum	11,25	8,799	5,049	8,438
	Maximum	160,1	135,9	25,96	40,32
