# Supplementary material for: Dynamics of transcriptional (re)-programming of syncytial nuclei in developing muscles
Source: BMC Biol. 2017 Jun 9;15:48. doi: 10.1186/s12915-017-0386-2 (PMC5466778; doi:10.1186/s12915-017-0386-2)
Supplement: Supplementary file 10 — FCM to FC/fibre programme conversion: dynamics of col + duf and col + sns transcription in a growing DA3 muscle. The number of nuclei and the number of col and duf or col and sns transcription dots were counted in wt embryos, using double FISH with col and duf or col and sns intronic probes coupled with Col and DAPI. The DA3 muscle was identified with the Col staining; the number of nuclei in the DA3 was counted with the DAPI staining. DAPI staining was also used to identify nuclei with only one or two transcription dots. For each muscle and stage, the mean number of dots (or nuclei) ± standard deviation, and minimum and maximum numbers of dots (or nuclei) are given (n = 12). (PDF 163 kb) [file 12915_2017_386_MOESM10_ESM.pdf]

Table S7: FCM to FC/fibre programs conversion: dynamics of *col* + *duf* and *col* + *sns* transcription in a growing DA3 muscle.

			stage 12	stage 13	stage 14	stage 15	stage 16
number of nuclei		Mean	1,00	2,67	6,75	9,50	10,92
		Std. Dev.	0,00	0,65	1,06	1,24	0,90
		Maximum	1	3	9	11	12
		Minimum	1	1	5	8	10
copropagation *col* and *duf*	*col*[i]	Mean	1,00	1,50	4,75	5,75	5,50
		Std. Dev.	0,00	0,67	1,055	1,29	1,00
		Maximum	1	3	7	8	7
		Minimum	1	1	3	3	4
	duf[i]	Mean	0,83	2,00	4,08	2,75	1,83
		Std. Dev.	0,39	0,85	0,90	1,06	1,40
		Maximum	1	3	5	5	4
		Minimum	0	1	2	1	0
	col[i] + *duf*[i]	Mean	0,83	1,00	3	1,83	1,25
		Std. Dev.	0,39	0,43	0,7385	0,83	1,22
		Maximum	1	2	4	3	3
		Minimum	0	0	2	1	0
copropagation *col* and *sns*	*col*[i]	Mean	1,08	2,17	4,83	5,67	n.d.
		Std. Dev.	0,29	0,72	0,83	1,44	
		Maximum	2	3	6	8	
		Minimum	1	1	3	3	
	sns[i]	Mean	0,00	0,08	0,50	0,00	n.d.
		Std. Dev.	0,00	0,29	0,52	0,00	
		Maximum	0	1	1	0	
		Minimum	0	0	0	0	
	col[i] + *sns*[i]	Mean	0,00	0,00	0,00	0,00	n.d.
		Std. Dev.	0,00	0,00	0,00	0,00	
		Maximum	0	0	0	0	
		Minimum	0	0	0	0	
