# Supplementary material for: Dynamics of transcriptional (re)-programming of syncytial nuclei in developing muscles
Source: BMC Biol. 2017 Jun 9;15:48. doi: 10.1186/s12915-017-0386-2 (PMC5466778; doi:10.1186/s12915-017-0386-2)
Supplement: Supplementary file 13 — Relative mRNA levels of duf and realisation genes. Relative levels of duf, Pax, mspo, kon and Con mRNA in DA3 and DT1 muscles were measured in wt embryos using FISH with exonic probes, and β3-Tub to visualise the muscle shape. The FISH mean intensity ± standard deviation is given for each muscle (n = 30). (PDF 7 kb) [file 12915_2017_386_MOESM13_ESM.pdf]

**Table S9: mRNA relative expression levels of *duf* and realisation genes.**

| | DA3 | DT1 |
|---|---|---|
| *duf* | 234.3 ± 24.8 | 240.2 ± 24.89 |
| *Pax* | 81.35 ± 10.77 | 108.8 ± 13.57 |
| *mspo* | 97 ± 7.99 | 270 ± 16.12 |
| *kon* | 203.4 ± 12.45 | 322.9 ± 17.98 |
| *Con* | 235.3 ± 13.58 | 370.9 ± 22.39 |
