# Supplementary material for: Dynamics of transcriptional (re)-programming of syncytial nuclei in developing muscles
Source: BMC Biol. 2017 Jun 9;15:48. doi: 10.1186/s12915-017-0386-2 (PMC5466778; doi:10.1186/s12915-017-0386-2)
Supplement: Supplementary file 14 — Numbers of nuclei and number of transcriptional dots of duf and realisation genes in DA3 and DT1 muscles at embryonic stages 12 to 16. The numbers of nuclei and transcriptional dots in the DA3 and DT1 muscles were counted in col LCRM -moeGFP; S59-mcd8GFP embryos using FISH with intronic probes, coupled with GFP and Topro staining. For each muscle and stage, the mean number of dots (or nuclei) ± standard deviation, and minimum and maximum numbers of dots (or nuclei) are given (n = 20). The same samples were also used for Additional files 15 and 16: Tables S11 and S12. (PDF 174 kb) [file 12915_2017_386_MOESM14_ESM.pdf]

Table S10: Numbers of nuclei and number of transcriptional dots of *duf* and realisation genes in DA3 and DT1 muscles at embryonic stages 12 to 16.

		stage 12		stage 13		stage 14		stage 15		stage 16	
		**DA3**	**DT1**	**DA3**	**DT1**	**DA3**	**DT1**	**DA3**	**DT1**	**DA3**	**DT1**
number	Mean	1.10	1.10	2.50	2.10	6.45	5.40	10.7	8.60	11.65	9.25
of nuclei	Std. Dev.	0.31	0.31	0.61	0.64	1.19	1.43	1.08	1.10	0.93	1.07
	Minimum	1	1	1	1	4	3	9	7	10	7
	Maximum	2	2	3	3	9	7	13	11	13	11
duf^{i}	Mean	1.00	0.75	2.20	1.85	2.55	2.05	1.95	1.80	0.10	0.10
	Std. Dev.	0.64	0.72	0.77	0.81	1.64	1.19	1.10	1.15	0.31	0.45
	Minimum	0	0	1	1	0	0	0	0	0	0
	Maximum	2	2	3	3	6	4	4	4	1	2
Pax^{i}	Mean	0.05	0.05	0.40	0.30	1.05	0.90	2.35	2.40	0.50	0.60
	Std. Dev.	0.22	0.22	0.60	0.66	1.10	1.02	1.53	0.88	0.83	0.88
	Minimum	0	0	0	0	0	0	0	0	0	0
	Maximum	1	1	2	2	3	3	5	4	3	3
mspo^{i}	Mean	0.00	0.05	0.90	0.45	1.35	2.05	1.75	3.25	0.80	1.00
	Std. Dev.	0.00	0.22	0.79	0.60	1.18	1.32	1.21	1.74	1.15	1.17
	Minimum	0	0	0	0	0	0	0	1	0	0
	Maximum	0	1	2	2	5	5	4	6	4	3
kon^{i}	Mean	0.70	0.35	0.95	0.75	2.15	3.30	1.30	4.35	0.30	0.40
	Std. Dev.	0.47	0.59	0.69	0.64	1.09	1.66	1.22	2.08	0.57	0.60
	Minimum	0	0	0	0	1	1	0	1	0	0
	Maximum	1	2	2	2	6	7	4	10	2	2
Con^{i}	Mean	0.00	0.00	0.30	1.25	0.45	3.55	0.60	3.95	0.85	1.50
	Std. Dev.	0.00	0.00	0.47	0.91	0.69	1.43	0.68	1.61	0.75	1.28
	Minimum	0	0	0	0	0	0	0	1	0	0
	Maximum	0	0	1	3	2	6	2	7	2	4
