# Supplementary material for: Dynamics of transcriptional (re)-programming of syncytial nuclei in developing muscles
Source: BMC Biol. 2017 Jun 9;15:48. doi: 10.1186/s12915-017-0386-2 (PMC5466778; doi:10.1186/s12915-017-0386-2)
Supplement: Supplementary file 15 — Integrated density of transcriptional dots of duf and realisation genes in DA3 and DT1 at stage 12 to 16. For each muscle and stage, the mean intensity of transcriptional dots in the DA3 and DT1 muscles ± standard deviation, and the minimum and maximum intensity are given for each probe (n = 20). Same embryo samples as in Additional files 14 and 16: Tables S10 and S12. (PDF 169 kb) [file 12915_2017_386_MOESM15_ESM.pdf]

Table S11: Integrated density of transcriptional dots of *duf* and realisation genes in DA3 and DT1 at stage 12 to 16.

		stage 12		stage 13		stage 14		stage 15		stage 16	
		**DA3**	**DT1**	**DA3**	**DT1**	**DA3**	**DT1**	**DA3**	**DT1**	**DA3**	**DT1**
duf[i]	Mean	54.62	75.22	138.20	177.10	89.74	101.50	63.69	79.91	19.04	30.72
	Std. Dev.	34.03	58.85	135.20	181.60	97.94	106.40	68.62	78.16	1.22	0.61
	Minimum	8.66	10.39	8.66	8.66	8.66	8.66	9.23	8.66	18.18	30.29
	Maximum	130.10	189.80	637.00	741.10	384.30	384.30	255.90	282.40	19.91	31.16
Pax[i]	Mean	45.00	60.00	66.29	56.17	66.19	86.61	71.02	72.23	92.33	74.33
	Std. Dev.	0.00	0.00	24.01	17.26	28.04	46.84	30.77	38.06	26.37	16.83
	Minimum	45.00	60.00	41.00	41.00	40.00	40.00	40.00	40.00	61.00	60.00
	Maximum	45.00	60.00	107.00	89.00	150.00	202.00	203.00	237.00	145.00	115.00
mspo[i]	Mean	n.a	123.00	88.50	86.00	90.96	114.20	96.94	103.70	67.75	76.95
	Std. Dev.	n.a	0.00	15.07	24.58	26.69	40.01	26.61	38.83	8.08	14.63
	Minimum	n.a	123.00	61.00	60.00	60.00	60.00	63.00	60.00	60.00	61.00
	Maximum	n.a	123.00	109.00	128.00	168.00	211.00	144.00	207.00	93.00	115.00
kon[i]	Mean	95.93	112.40	100.40	98.07	107.80	110.70	103.40	111.80	104.50	90.50
	Std. Dev.	24.73	34.90	29.07	24.09	41.58	39.87	27.87	39.62	36.03	38.99
	Minimum	64.00	69.00	60.00	63.00	60.00	60.00	61.00	60.00	71.00	63.00
	Maximum	135.00	161.00	160.00	151.00	226.00	255.00	177.00	217.00	160.00	182.00
Con[i]	Mean	n.a	n.a	52.17	102.50	39.00	95.93	36.58	80.06	50.18	81.57
	Std. Dev.	n.a	n.a	26.98	55.83	16.08	47.86	14.45	48.46	28.34	49.09
	Minimum	n.a	n.a	23.00	20.00	20.00	21.00	20.00	20.00	20.00	20.00
	Maximum	n.a	n.a	87.00	200.00	68.00	214.00	66.00	208.00	124.00	185.00
