# Supplementary material for: Dynamics of transcriptional (re)-programming of syncytial nuclei in developing muscles
Source: BMC Biol. 2017 Jun 9;15:48. doi: 10.1186/s12915-017-0386-2 (PMC5466778; doi:10.1186/s12915-017-0386-2)
Supplement: Supplementary file 16 — Total integrated density per fibre of duf and realisation genes at stages 12 to 16. For each stage, the total intensity of transcriptional dots in the DA3 and DT1 muscles and the average of total intensity ± SEM are given (n = 20). Same embryo samples as Additional files 14 and 15: Tables S10 and S11. (PDF 156 kb) [file 12915_2017_386_MOESM16_ESM.pdf]

Table S12: Total Integrated density per fibre of *duf* and realisation genes at stage 12 to 16.

		stage 12		stage 13		stage 14		stage 15		stage 16	
		**DA3**	**DT1**	**DA3**	**DT1**	**DA3**	**DT1**	**DA3**	**DT1**	**DA3**	**DT1**
duf^{i}	Mean	51.88	56.41	290.2	327.7	228.8	258.2	124.2	147.2	1.904	3.072
	SEM	10.3	15.03	54.25	50.96	50.64	59.37	23.55	36.29	1.312	3.072
Pax^{i}	Mean	2.25	3	28.3	16.85	69.5	77.95	166.9	173.4	41.55	44.6
	SEM	2.25	3	9.253	8.064	15.9	21.00	27.01	18.84	16.31	14.47
mspo^{i}	Mean	0	6.15	79.65	38.7	122.8	234.2	169.7	337.7	54.2	76.95
	SEM	0	6.15	16.55	11.5	22.03	39.38	25.99	47.72	18.28	19.97
kon^{i}	Mean	67.15	39.35	96.65	73.55	231.7	359.9	134.5	480.9	31.35	36.2
	SEM	11.07	14.35	16.57	15.15	31.89	41.83	27.75	60.21	14.14	12.42
Con^{i}	Mean	0	0	15.65	128.1	17.55	335.8	21.95	313.3	40.3	122.4
	SEM	0	0	6.298	26.52	5.952	38.61	6.012	37.46	9.601	25.97
