# Supplementary material for: Dynamics of transcriptional (re)-programming of syncytial nuclei in developing muscles
Source: BMC Biol. 2017 Jun 9;15:48. doi: 10.1186/s12915-017-0386-2 (PMC5466778; doi:10.1186/s12915-017-0386-2)
Supplement: Supplementary file 18 — Percentage of dots and nuclei localised in the antero-ventral, central and postero-dorsal DA3 subdomains. Spatial coordinates of nuclei and transcriptional dots were acquired on col LCRM -moeGFP late stage 14 embryos stained for GFP and Col. FISH were with intronic probes. For each gene, the percentage of dots localised in antero-ventral (Relative Delta Y < –1), postero-dorsal (Relative Delta X > 1) or central (Relative Delta X < 1 and Relative Delta Y > –1) position are shown; 25 muscles were analysed for each condition, the corresponding total number of spots (or nuclei) is indicated. (PDF 145 kb) [file 12915_2017_386_MOESM18_ESM.pdf]

Table S13: Percentage of dots and nuclei localised in the antero-ventral, central and postero-dorsal DA3 sub-domains.

	Antero-Ventral (%)	Central (%)	Postero-Dorsal (%)	number of spots
col[i]	35.15	23.64	41.21	165
Mhc[i]	32.71	37.17	30.11	269
duf[i]	27.27	31.82	40.91	66
mspo[i]	39.51	28.40	32.10	81
kon[i]	38.78	32.65	28.57	49
Con[i]	15.63	68.75	15.63	32
Nuclei	36.36	30.58	33.60	242
