# Supplementary material for: Dynamics of transcriptional (re)-programming of syncytial nuclei in developing muscles
Source: BMC Biol. 2017 Jun 9;15:48. doi: 10.1186/s12915-017-0386-2 (PMC5466778; doi:10.1186/s12915-017-0386-2)
Supplement: Supplementary file 19 — Supplementary Material and Methods. 5’-3’ sequence of the oligonucleotide primers used for making probes for FISH. (PDF 527 kb) [file 12915_2017_386_MOESM19_ESM.pdf]

Oligonucleotide primers used for making FISH RNA probes:

col^{i}, *mspo* and *Pax* RNA probes were synthetized as previously described [1, 2].

S59^{i}	5’-GGAGATTTGGGCACGATAAG-3’	+ 5’-CCAGAGCAAGAGAGGTCGG-3’
Kr^{i}	5’-CGCACAAACGCGAAGT-3’	+ 5’-GAGAGTGTTGGCCAATGTGCTC-3’
duf^{i}	5’-GCCAACGAGCAGCTGATGG-3’	+ 5’-GTGTCGTCGTGTGTTTGTGC-3’
sns^{i}	5’-CATCGCCATCTGTTTGTGTG-3’	+ 5’-GTTTGCTGTGTTTACAGTTCG-3’
kon	5’-CGTGTTATTGACTAACCGAAG-3’	+ 5’-CCAAGTCTGAACGAGCAATC-3’
kon^{i}	5’-GACGACAACGAGAAAAGTGC-3’	+ 5’-CTGTCGCAGCCATGAAAGG-3’
mspo^{i}	5’-CTATGGATACCAAGTACTTCG-3’	+ 5’-GTTGGTCTAGGCTTGTGTATC-3’
Pax^{i}	5’-GCAACAACAACAGCCAAACC-3’	+ 5’-GAGCGTGACGTTCTGATAAC-3’
Con	5’-CTATAGCGGACCGATGGAC-3’	+ 5’-CTGGTCAGCACATTGATCTG-3’
Con^{i}	5’-CCATTAATCCCGTTGTCATAAC-3’	+ 5’-GCGCTCGGAAATGCGCTG-3’

The corresponding PCR products were cloned in pGemTeasy, and the antisense RNA probes were transcribed *in vitro*, using T7 or SP6 RNA polymerase.

Mhc	5’-GATCAGGAGATCTCTGGCCTGAAGAAG-3’ +<br>5’-ccgaattctaatacgactcactatagggagaCTTCAGCTTGTTGAGCTGATCAACCTG-3’
Mhc^{i}	5’-CCAAGTTCCGTTTCAAAATCCGATC-3’ +<br>5’-ccgaattctaatacgactcactatagggagaCGTATCGAACAGCGTTATGGTTAGTAGAC-3’

The corresponding antisense RNAs probes were transcribed *in vitro* with T7 RNA polymerase, using PCR templates. The T7 sequence is in minus characters.

1. Crozatier M, Vincent A: **Requirement for the Drosophila COE transcription factor Collier in formation of an embryonic muscle: transcriptional response to notch signalling**. *Development* 1999, **126**(7):1495-1504.
2. Bataillé L, Delon I, Da Ponte JP, Brown NH, Jagla K: **Downstream of identity genes: muscle-type-specific regulation of the fusion process**. *Dev Cell* 2010, **19**(2):317-328.
